# De novo production of bioactive sesterterpenoid ophiobolins in *Saccharomyces cerevisiae* cell factories

**DOI:** 10.1186/s12934-024-02406-0

**Published:** 2024-05-06

**Authors:** Caizhe Zhang, Jun Wu, Qing Sun, Shuaishuai Ding, Hua Tao, Yuhua He, Hui Qiu, Bei Shu, Dongqing Zhu, Hengcheng Zhu, Kui Hong

**Affiliations:** 1 Department of Radiation and Medical Oncology, Key Laboratory of Combinatorial Biosynthesis and Drug Discovery, School of Pharmaceutical Sciences, Zhongnan Hospital, Ministry of Education, Wuhan University, Wuhan, 430071 China; 2 Department of Urology, Renmin Hospital of Wuhan University, No. 238 Jie-Fang Avenue, Wuhan, 430060 China

**Keywords:** Sesterterpenoid, Ophiobolin, *Saccharomyces cerevisiae*, Metabolic engineering, Whole-cell transformation

## Abstract

**Background:**

Sesterterpenoids are rare species among the terpenoids family. Ophiobolins are sesterterpenes with a 5-8-5 tricyclic skeleton. The oxidized ophiobolins exhibit significant cytotoxic activity and potential medicinal value. There is an urgent need for large amounts of ophiobolins supplication for drug development. The synthetic biology approach has been successfully employed in lots of terpene compound production and inspired us to develop a cell factory for ophiobolin biosynthesis.

**Results:**

We developed a systematic metabolic engineering strategy to construct an ophiobolin biosynthesis chassis based on *Saccharomyces cerevisiae*. The whole-cell biotransformation methods were further combined with metabolic engineering to enhance the expression of key ophiobolin biosynthetic genes and improve the supply of precursors and cofactors. A high yield of 5.1 g/L of ophiobolin F was reached using ethanol and fatty acids as substrates. To accumulate oxidized ophiobolins, we optimized the sources and expression conditions for P450-CPR and alleviated the toxicity of bioactive compounds to cells through PDR engineering. We unexpectedly obtained a novel ophiobolin intermediate with potent cytotoxicity, 5-hydroxy-21-formyl-ophiobolin F, and the known bioactive compound ophiobolin U. Finally, we achieved the ophiobolin U titer of 128.9 mg/L.

**Conclusions:**

We established efficient cell factories based on *S. cerevisiae*, enabling de novo biosynthesis of the ophiobolin skeleton ophiobolin F and oxidized ophiobolins derivatives. This work has filled the gap in the heterologous biosynthesis of sesterterpenoids in *S. cerevisiae* and provided valuable solutions for new drug development based on sesterterpenoids.

**Supplementary Information:**

The online version contains supplementary material available at 10.1186/s12934-024-02406-0.

## Background

Sesterterpenoids are rare species in the terpene family, less than 1.5% among the 182,672 terpenoid molecules (http://terokit.qmclab.com/). Ophiobolins are sesterterpene compounds with 5-8-5 tricyclic carbon skeleton and exhibit a broad spectrum of bioactivities, including prominent antitumor and antibacterial activities [1]. Our previous study demonstrated that 6-epi-ophiobolin G exerts potent antitumor effects on triple-negative breast cancer, which is considered the most challenging subtype among all breast cancers [2]. Hence, efficient synthesis of bioactive ophiobolins is crucial in supporting the pharmacological investigation of these compounds and ensuring an adequate supply of candidate drugs. In the field of chemical synthesis, numerous researchers have conducted extensive studies on the chemical synthesis of ophiobolins over several decades. However, the total synthetic routes remain highly complex, and the final yields have consistently been suboptimal [3–6]. As for biosynthesis, using native strains for production is typically hindered by the complexity of metabolites, difficulties in separation, and challenges such as a long fermentation period and complex genetic manipulation [7]. In recent years, with the development of synthetic biology, constructing cell factories for producing valuable terpenoid compounds has emerged as a more efficient alternative, including carotenoids and squalene used as healthcare products [8–10], vincristine and artemisinin as natural pharmaceuticals [11–13], and limonene employed in fragrances [14]. Using synthetic biology approaches for heterologous biosynthesis of ophiobolins represents an attractive option.

**Fig. 1:**
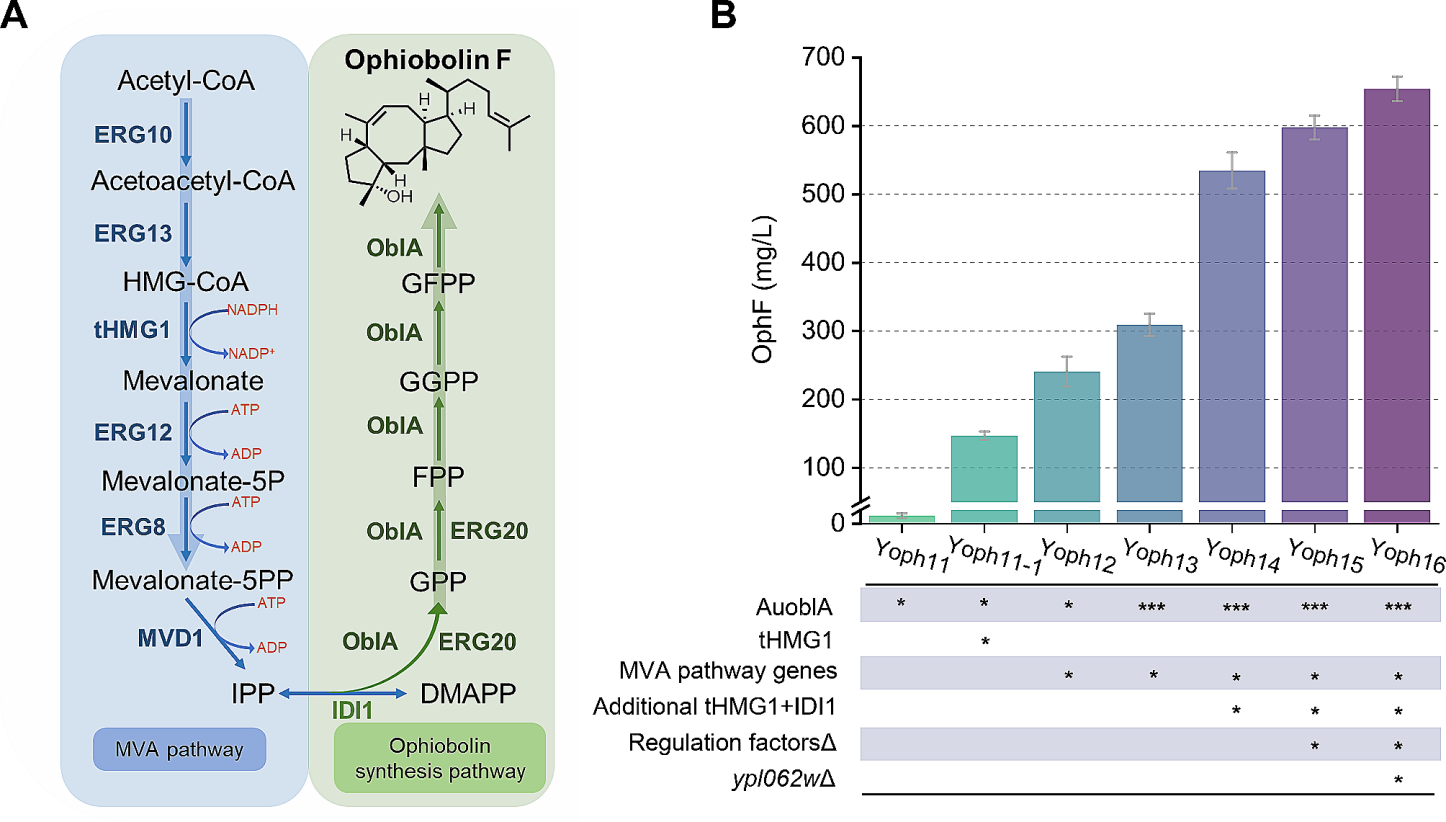
Improving ophiobolin F (OphF) biosynthesis by enhance the terpenoid precursors supply. (**A**) OphF synthetic pathway in *S. cerevisiae*. Enzymes marked in blue and green color are overexpressed. ERG10, acetyl-CoA acetyltransferase; ERG13, 3-hydroxy-3-methylglutaryl-CoA (HMG-CoA) synthase; tHMG1, truncated HMG-CoA reductase; ERG12, mevalonate kinase; ERG8, phosphomevalonate kinase; MVD1, mevalonate pyrophosphate decarboxylase; IDI1, isopentenyl diphosphate; ERG20, farnesyl pyrophosphate synthetase; OblA, ophiobolin F synthase; (**B**) Titer of OphF produced by engineered strains. Each * represents one genetic manipulation. The cells were cultivated in YPD-Z medium for 96 h. Bar heights represent the averages of three biological replicates, and error bars represent standard deviation

Previous investigations have identified the biosynthetic gene cluster of ophiobolins and the post-modification processes of ophiobolin F (OphF). In summary, acetyl-CoA is converted to the terpene precursor isopentenyl pyrophosphate (IPP) and dimethylallyl pyrophosphate (DMAPP) via the MVA pathway. Subsequently, the ophiobolin synthase OblA utilizes IPP/DMAPP for chain elongation, resulting in the production of GFPP (C25), which is then cyclized to form the ophiobolin skeleton OphF (Fig. 1A). Finally, OphF is catalyzed by P450 enzyme and FAD-dependent oxidase to produce different structural forms of ophiobolins [7, 15–17]. The availability of acetyl-CoA and the flux through the MVA pathway are critical factors for the synthesis of OphF. However, there have been limited efforts to engineer the de novo microbial biosynthesis of ophiobolins. Our earlier studies harnessed *Escherichia coli* (*E. coli*) as the host to synthesize OphF, representing the first attempt to construct a high-yield chassis for bifunctional terpene synthase sesterterpenoids [18]. However, due to the inefficient precursor supply, the yield of OphF was only 150 mg/L, demonstrating lower production efficiency compared to native strains such as *Aspergillus ustus*. Furthermore, OphF does not possess biological activity, and most bioactive ophiobolins are generated by modifying OphF through cytochrome P450 enzymes and other post-synthetic enzymes [1]. As *E. coli* lacks a well-developed membrane system, it is not the optimal host for expressing membrane-bound P450 enzymes. Our previous attempts to express the P450 enzyme for the post-modification of OphF in *E. coli* did not yield the desired oxidized products [19].

In contrast to prokaryotes, yeast, such as *Saccharomyces cerevisiae* (*S. cerevisiae*), possesses organelles like the endoplasmic reticulum and mitochondria, providing favorable conditions for the expression of enzymes localized to organelle membranes [20, 21]. Additionally, yeast has a more extensive protein post-modification capability, which is advantageous for the heterologous expression of P450 enzymes [11, 12]. Furthermore, as a Generally Recognized as Safe (GRAS) strain, *S. cerevisiae* is a more suitable host for producing potential therapeutic ophiobolins.

The biosynthesis of ophiobolins requires an abundant supply of building blocks. As OphF is derived from the cyclization of GFPP, which requires four IPP and one DMAPP [18]. Strategies such as enhancing the mevalonate (MVA) pathway and introducing IPP bypass pathways have effectively increased the supply of IPP/DMAPP [9, 22, 23]. Precursor acetyl-CoA and cofactor NADPH content are key limiting factors in terpene synthesis. Global metabolic reprogramming to enhance acetyl-CoA supply has been identified as a practical approach [24, 25]. Utilizing compartmentalization engineering to harness the abundant acetyl-CoA in mitochondria and peroxisomes has been widely reported [21, 26]. Addressing the issue of insufficient NADPH supply in the MVA pathway, optimization of the NADPH/NADP^+^ ratio can be performed by modifying key genes in the PPP pathway [14, 27, 28].

Industrial strains’ metabolic, gene regulatory, and signaling networks are complex. In fermentations, coordinating cell growth and product accumulation poses a major challenge [29]. Compared to conventional fermentation, whole-cell transformation allows a complete separation of the cell growth and product synthesis stages, thereby reducing interference from cellular metabolic bypass pathways and avoiding the generation of complex metabolic byproducts [30]. Research on de novo synthesis of terpenoids using whole-cell transformation is limited. Niu et al. [31] constructed the *E. coli* whole-cell biocatalysis system for the production of pinene, achieving a yield of 166.5 mg/L, which is 1.6-fold higher than the fermentation process, the higher cell density and the growth-arrested cells may be the primary reasons for high productivity.

This study aims to utilize *S. cerevisiae* to construct cell factories for the biosynthesis of bioactive ophiobolins and explore the potential of whole-cell biocatalysts system in terpenoid production. Initially, we enhanced the MVA pathway, optimized the synthetic pathways to achieve the de novo synthesis of OphF, and obtained a high-yield OphF chassis strain. Subsequently, we optimized the catalytic conditions of the whole-cell conversion system. Using ethanol as a substrate, we increased the metabolic flux from ethanol to acetyl-CoA and further enhanced acetyl-CoA supply through β-oxidation of fatty acids. Furthermore, by overexpressing NADH kinase POS5 to elevate intracellular NADPH levels, we yielded over 5 g/L of OphF in shake flasks, 33-fold higher than the reported maximum production (150 mg/L). The shake flask-level production of sesterterpene is the highest titer reported in *S. cerevisiae* to date and serves as an exemplary case of combining whole-cell biocatalysis with metabolic engineering. To obtain biologically active ophiobolins, we optimized the expression of the P450-CPR catalytic system and unexpectedly obtained a structurally novel ophiobolin molecule with strong cytotoxic activity, along with the known compound ophiobolin U (OphU) at the titer of 128.9 mg/L. Considering the toxicity of these compounds to yeast growth, we implemented transporter engineering, increasing the proportion of product efflux and raising the yield of OphU.

## Materials and methods

### Media, culture conditions, and materials

*S. cerevisiae* strains were cultured at 30 ℃ in YPD-Z medium (1% yeast extract, 2.5% peptone, 2% glucose, 0.2% MgSO_4_ and 0.8% KH_2_PO_4_, pH = 6.0). Synthetic drop-out (SD) medium lacking leucine, or/and uracil, was used for the selection of recombinants containing the corresponding marker(s). Agar (1.8% w/v) added to SD medium for preparing solid medium used in screening transformants. The solid SD complete medium containing 1 mg/mL of 5-fluoroorotic acid was used for the elimination of the URA3 marker in the strains. For shake-flask cultures, single colonies were picked and inoculated into a tube containing 5 mL YPD-Z medium for overnight cultivation at 30 °C and 220 rpm, then inoculated into a 250 mL shake flask containing 50 mL YPD-Z medium with an initial OD_600_ of 0.2, and grown under the same condition for 96 h and overlaid with 10% dodecane to trap ophiobolins.

Restriction enzymes and T4 DNA ligase were purchased from New England Biolabs (Beverly, MA, USA). Phanta HS Super-Fidelity DNA Polymerase and One Step Cloning Kit were purchased from Vazyme Biotech Co., Ltd. (Nanjing, China). DNA gel purification and plasmid extraction kits were purchased from Omega Bio-Tek (Norcross, GA, USA). All primer synthesis, codon optimized genes synthesis and DNA sequencing were performed by TSINGKE Biotech (Beijing, China). All chemicals were purchased from Sangon Biotech (Shanghai, China) unless stated otherwise.

### Construction of plasmids and strains

*Escherichia coli* strain DH5α was used for the construction and propagation of plasmids. The key strains in this study were listed in Table 1. The flowchart of yeast strain construction is described in Additional file 1: Fig. S1. All primers and gRNA sequences are listed in Additional file 1: Table S1 and Table S2. The genomic DNA of CEN.PK2-1D was used as a template for amplifying native DNA fragments including promoters, endogenous genes, terminators and homologous arms. The codon optimized genes for *S cerevisiae* are listed in Additional file 1: Table S3. All donor DNAs for gene deletion and integration were generated by overlapping extension PCR or One Step Cloning Kit. The genes were integrated into the yeast genome using CRISPR/Cas9. All guide-RNAs (gRNAs) were designed by the CRISPRdirect webtool [32] (https://crispr.dbcls.jp), and the construction method of the gRNA plasmid refers to the Zhang et al. [33]. Yeast transformation was carried out using the LiAc/SS carrier DNA/PEG method [34].

**Table 1:** Saccharomyces cerevisiae strains used in this study

Strain name	Genotype	Resource
CEN.PK2-1D	MATalpha; his3D1; leu2-3_112; ura3-52; trp1-289; MAL2-8c; SUC2	EUROSCARF
Yoph1	CEN.PK2-1D, *gal80*Δ	This work
Yoph11	Yoph1, ChrI-1:: P_GAL10_-*AuoblA*-T_ADH1_	This work
Yoph11-1	Yoph11, ChrVI-1:: P_GAL10_- tHMG1- T_HMG1_	This work
Yoph12	Yoph11, ChrIII-1:: T_HMG1_-*tHMG1*-P_GAL10_-P_GAL1_-*ERG13-T*_*ERG13*_; ChrIV-1:: T_MVD1_-*MVD1-*P_GAL10_-P_GAL1_-*ERG20-T*_*ERG20*_; ChrXII-1:: T_ERG8_-*ERG8-*P_GAL10_-P_GAL1_-*ERG10-T*_*ERG10*_; ChrXV-1:: T_IDI1_-*IDI1-*P_GAL10_-P_GAL1_-*ERG12-T*_*ERG12*_	This work
Yoph13	Yoph12, ChrVI-1:: T_HMG1_-*tHMG1*-P_GAL10_-P_GAL1_-*IDI1*-T_IDI1_	This work
Yoph14	Yoph13, *dpp1*Δ:: P_GAL10_-*AuoblA*-T_ADH1_; *lpp1*Δ:: P_GAL10_-*AuoblA*-T_ADH1_	This work
Yoph15	*dos2*Δ; *ynr063wΔ*; *ygr259cΔ*; *vba5Δ*; *yer134cΔ*	This work
Yoph16	Yoph15, *ypl062wΔ*	This work
Yoph18	Yoph15, *dos2Δ*:: P_GAL2_-*tPOS5*-T_ADH1_	This work
Yoph19	Yoph18, ChrX-1:: (P_GAL7_-*ADH2*-T_TDH1_) + (P_PGK1_-*ACS1*-T_ACS1_); vba5Δ:: P_TEF1_-*ALD6*-T_ADH1_	This work
Yoph20	Yoph19, ChrII-1:: P_GAL2_-*CAT2pex-T*_*PDC1*_	This work
Yoph31	Yoph14, *ypl062wΔ*:: P_GAL1_-*AuoblB*-T_CYC1_	This work
Yoph32	Yoph14, *ypl062wΔ*:: P_GAL1_-*AcoblB*-T_CYC1_	This work
Yoph33	Yoph14, *ypl062wΔ*:: P_GAL1_-*BmoblB*-T_CYC1_	This work
Yoph34	Yoph33, ChrVII-1:: P_TDH3_-*AuoblC*-T_CYC1_	This work
Yoph35	Yoph34, ChrXI-1:: P_GAL1_-*BmoblB*-T_CYC1_	This work
Yoph36	Yoph35, ChrIV-2:: P_TEF1_- *Au*_*9779*_*CPR*-T_PRM9_	This work
Yoph37	Yoph35, ChrIV-2:: P_TEF1_- *Au*_*674*_*CPR*-T_PRM9_	This work
Yoph38	Yoph36, ChrIV-2:: (P_TEF1_- *Au*_*9779*_*CPR*-T_PRM9_) + (P_PGK1_-*AuCYB5*-T_IDP1_)	This work
Yoph39	Yoph35, ChrIV-2:: P_TEF1_- *NCP1*-T_PRM9_	This work
Yoph40	Yoph36, ChrX-2:: P_TDH3_-*PDR1*-T_CYC1_	This work
Yoph41	Yoph36, ChrX-2:: P_TDH3_-*PDR3*-T_CYC1_	This work
Yoph42	Yoph36, ChrX-2:: P_TDH3_-*PDR5*-T_CYC1_	This work
Yoph43	Yoph36, ChrX-2:: P_TDH3_-*PDR10*-T_CYC1_	This work
Yoph44	Yoph36, ChrX-2:: P_TDH3_-*SNQ2*-T_CYC1_	This work

### Whole-cell transformation for ophiobolin F production

A single colony was picked and inoculated into a tube containing 5 mL YPD-Z medium for overnight cultivation at 30 °C and 220 rpm, then inoculated into a 250 mL shake flask containing 50 mL YPD-Z medium with an initial OD_600_ of 0.2. After 8 h of glucose depletion, yeast cells were harvested by centrifugation. The collected cells were washed once with 0.9% NaCl and centrifuged again. Then suspended in 0.05 mol/L phosphate buffer with 2 g/L MgSO_4_ (pH 6.0), resulting in a final cell concentration of OD_600_ = 100. Subsequently, add carbon source to initiate the production of OphF. The catalysis system is carried out at 30 °C and 200 rpm, with the reaction mixture covered by 10% dodecane. The pH of the catalysis system was controlled between 5.5 and 6.0 using HCl and NaOH.

### Spot dilution growth assay

Single colonies were inoculated into 5 mL YPD-Z medium for overnight cultivation at 30 °C and 220 rpm, then diluted to an OD_600_ of 2.0 in fresh medium. 4 µL of tenfold serial dilutions were spotted onto YPD-Z plates or YPD-Z with 150 µM of OphU and incubated at 30 ℃ for 2–3 days.

### Analytical methods

#### Separation and analysis of ophiobolins

The cells and dodecane phase were separated by centrifugation at 12,000 rpm for 5 min. The extracellular excreted ophiobolins were captured by dodecane, and diluted with ethyl acetate to an appropriate concentration for detection. The cells were suspended in acetone to extract the ophiobolins not excreted, then mixed on a vortex for 5 min and ultrasound extracted for 10 min, followed by centrifugation to collect the supernatant for analysis. Liquid chromatography (SHIMADZU LC-20 AT) equipped with an Eclipse XDB-C18 column (250 × 4.6 mm, 5 μm, Agilent Technologies, Germany) was used for the quantitative analysis of ophiobolins. The quantification of yields is based on the integration of UV signals. The mobile phase was 100% acetonitrile, with a flow rate of 1 mL/min for 30 min, and the column temperature was 25 °C. Detection wavelengths were set at 190 nm for OphF and 240 nm for OphU and 5-hydroxy-21-formyl-Ophiobolin F. The retention times of OphF, 5-hydroxy-21-formyl-Ophiobolin F, and OphU were 17.5, 5.5, and 4.7 min, respectively (Additional file 1: Figure S4). The ophiobolins isolated by semi-preparative HPLC (Agilent 1260 Infinity, Agilent Technologies, Germany) equipped with an Eclipse XDB-C18 (250 × 9.4 mm, 5 μm, Agilent Technologies, Germany). The mobile phase was 100% acetonitrile, with a flow rate of 3 mL/min for 30 min, and the column temperature was 25 °C.

#### Structure elucidation of ophiobolins

1D and 2D NMR data were recorded in CDC_l3_ using a NEO-600 spectrometer with 1 H-NMR spectrum at 600 MHz and ^13^C-NMR spectrum at 150 MHz, separately. Chemical shifts are referenced to solvent peaks of CDC_l3_ (δH 7.26 ppm, δC 77.16 ppm). HR-ESIMS spectra were recorded on a quadrupole time-of-flight mass instrument (Agilent). ECD spectra were measured using a Chirascan V100 spectropolarimeter. The structural elucidation of OphU and 5-OH-21-CHO-ophF are described in the supplementary materials.

#### Determination of the NADPH/NADP^+^ ratio and glucose concentration

The NADPH and NADP^+^ content of cells in whole-cell transformation was evaluated using an NADPH/NADP^+^ assay kit (#S0179, Beyotime, Shanghai, China) according to the manufacturer’s instructions. The glucose content of medium was evaluated using glucose assay kit (#60408ES60, Yeasen Biotechnology, Shanghai, China) according to the manufacturer’s instructions.

### Cytotoxicity assay

Evaluate the in vitro anti-proliferative activity of compounds against MDA-MB-231 (human TNBC cell line), Siha (human cervical cancer cell line), and A2780 (A2780 ovarian cancer cell line) using the MTT assay. Use cisplatin and Adriamycin as positive controls. All cell lines are maintained in our laboratory. Cells are seeded in a 96-well plate at a density of 8,000 cells per well with 10% PBS and cultured for 24 h at 37 °C in 5% CO_2_ incubator. MDA-MB-231 and Siha were cultured in Dulbecco’s Modified Eagle Medium (DMEM, HyClone, Logan, Utah, USA) and A2780 cells in Roswell Park Memorial Institute 1640 medium (RPMI 1640, HyClone, Logan, Utah, USA). Subsequently, cells are treated with different concentrations of compounds for 48 h. This experiment uses DMSO (1 µL/ml or less) as the control culture vehicle. After 48 h of compound treatment, fresh cell culture medium is replaced, and 15 µL of MTT solution is added to each well (total volume per well is 100 µL). The cells are then incubated for 4 h at 37 °C in 5% CO_2_ incubator. Subsequently, the absorbance values are measured at 570 nm wavelength using a microplate reader. The concentration at which growth is reduced by 50% is determined as the half-maximal inhibitory concentration (IC_50_).

## Results and discussion

### Engineering *S. cerevisiae* for the production of ophiobolin F

The biosynthetic pathway of OphF has been clarified [15, 16]. OblA (AcOS), responsible for the ophiobolin skeleton synthesis, consists of two functional domains: a C-terminal prenyltransferase for geranylgeranyl pyrophosphate (GFPP) synthesis and an N-terminal cyclase [16]. In vitro enzyme studies demonstrated that OblA (Au8003) can independently carry out both chain elongation and cyclization reactions using dimethylallyl pyrophosphate (DMAPP), geranyl pyrophosphate (GPP), farnesyl pyrophosphate (FPP), and geranylgeranyl pyrophosphate (GGPP) as initial substrates, ultimately producing ophF [15] (Fig. 1A).

In order to avoid the utilization of high-cost galactose, the regulatory gene *gal80* of the *S. cerevisiae* CEN.PK2-1D strain was knocked out to obtain the Yoph1 strain, in which the gal promoter was only regulated by glucose concentration without the need for additional induction with galactose [35]. Subsequently, the ophiobolin synthase gene *AuoblA* from *A. ustus* 094102 was introduced into Yoph1, resulting in the Yoph11 strain. The OphF titer of the resultant Yoph11 strain reached 7.5 mg/L (Fig. 1B). OblA can directly synthesize OphF using C5 units, including DMAPP and isopentenyl pyrophosphate (IPP), derived from the MVA pathway [15, 16, 18]. An adequate supply of DMAPP/IPP precursors is crucial for accumulating the target product. HMG-CoA reductase (HMGR) is the rate-limiting enzyme in MVA pathway, with a structure at the N-terminus targeting the protein to the endoplasmic reticulum, and a catalytic active site at the C-terminus. Studies have shown that truncating the targeting structure at the N-terminus exposes the catalytic active center at the C-terminus to the cytoplasm, significantly increasing the catalytic activity of HMGR [14]. Based on the Yoph11 strain, the truncated HMG-CoA reductase tHMG1 was overexpressed, resulting in the Yoph11-1 strain. Overexpression of tHMG1 effectively increased the production of ophiobolin F (Fig. 1B). However, considering that the expression of a single copy of the tHMG1 gene may not maximize the supply of IPP/DMAPP, we subsequently overexpressed all genes in the MVA pathway to strengthen the precursor supplication.

The eight genes of the MVA pathway (*ERG10*, *ERG13*, *tHMG1*, *ERG12*, *ERG8*, *MVD1*, *IDI1*, *ERG20*) were overexpressed using the gal1,10 promoter to enhance the pathway. To improve gene manipulation efficiency, a multiplexed CRISPR-Cas9 system was used for the one-step chromosomal integration of these genes [33], resulting in the Yoph12 strain. The enhancement of the MVA pathway significantly increased the accumulation of OphF, reaching a yield of 240.5 mg/L (Fig. 1B).

In addition to tHMG1, IDI1 is another rate-limiting enzyme in the MVA pathway [14, 36], additional copy of these two genes were expressed, generating the Yoph13 strain with 309 mg/L OphF (Fig. 1B). With ample precursor supply, the expression of the *AuoblA* gene might become a limiting factor. Genes *lpp1* and *dpp1*, encoding phosphate phosphatases, and their knockout reduces the consumption of precursors such as FPP and GGPP [37, 38], thereby allowing more GFPP to be catalyzed by AuoblA into OphF. Therefore, based on Yoph13, the ORFs of *lpp1* and *dpp1* were knocked out, and *AuoblA* was integrated at each knockout site to obtain the Yoph14 strain, achieving a production yield of 531.7 mg/L of OphF (Fig. 1B). Trikka et al. developed a system to screen a collection of viable yeast deletion strains using a carotenoid-based screen for identifying genes favorable for terpenoid synthesis after deletion. They found that knocking out the regulator genes of *dos2, ynr063w, ygr259c, vba5, and yer134c* can enhance the production of carotenoid and sclareol without affecting the growth of the strain [39]. We knocked out the above five genes, and observed a 12.5% improvement in OphF production in Yoph15, reaching 597.4 mg/L (Fig. 1B). Additionally, *ypl062w* was identified as the core promoter of *ald6* and deletion of *ypl062w* effectively enhances the production of monoterpenes, sesquiterpenes, diterpenes, triterpenes, and tetraterpenes in brewing yeast, while reducing the accumulation of acetic acid. This is achieved by upregulating acetyl-CoA synthesis, the mevalonate (MVA) pathway, and the expression of exogenous genes, thereby strengthening the synthesis of exogenous terpenoids [40]. We also evaluated the effect of *ypl062w* gene knockout, resulting in the Yoph16 strain, which further increased OphF production to 661.3 mg/L (Fig. 1B).

### Optimizing the whole-cell biotransformation system for the production of ophiobolin F

Using resting cells for whole-cell biotransformation allows for a complete separation of cell growth and product accumulation, thereby minimizing interference from metabolic bypass pathways. This approach might contribute to efficient production in cell factories. Additionally, the transformation system is relatively simpler in composition than the culture medium, making separating and purifying the product more accessible. The substrate selection range is more extensive, enabling the addition of more suitable substrates tailored to different target pathways or products. Furthermore, removing essential nutrients (such as nitrogen sources and trace elements) leads to cellular growth cessation, resulting in higher yields since more carbon sources are used for product synthesis rather than biomass accumulation [30].

**Fig. 2:**
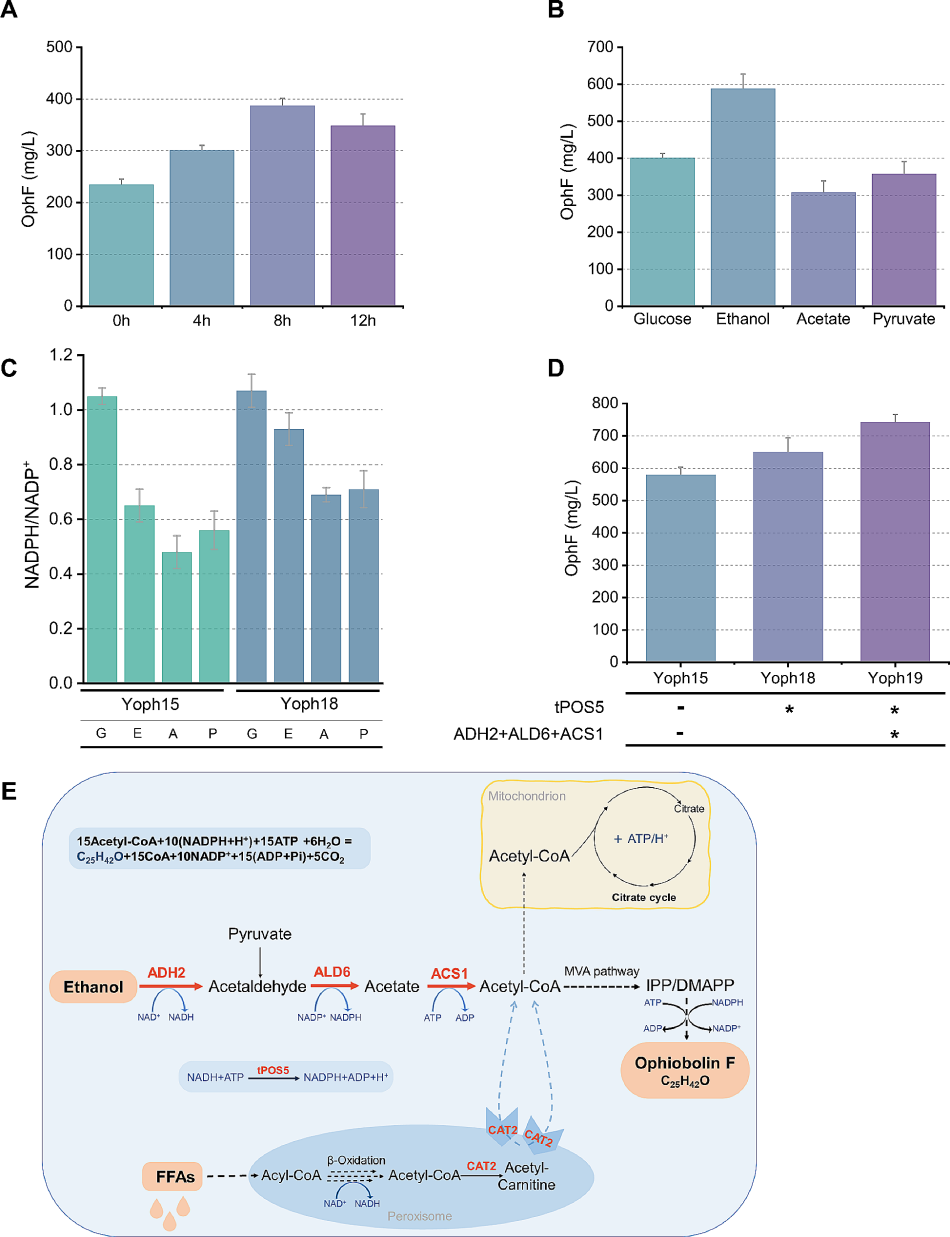
Whole-cell transformation for the synthesis of OphF. (**A**) The impact of different cultivation times on catalytic efficiency. 0 h, 4 h, 8 h, and 12 h respectively represent the time of continued cultivation after glucose depletion. (**B**) Comparison of adding different carbon sources during the whole-cell transformation process. (**C**) Intracellular NADPH/NADP^+^ content in different carbon sources and strains. G/E/A/P stands for glucose, ethanol, acetate, and pyruvate, respectively. (**D**) The impact of increasing the metabolic flux from acetyl-CoA to ethanol and supplying NADPH on the synthesis of OphF. (**E**) Schematic diagram of utilizing ethanol and fatty acids for OphF synthesis in whole-cell transformation. In the cell growth phase, YPD-Z medium was used, followed by centrifugation and washing before initiating whole-cell transformation, resulting in a final cell concentration of OD_600_ = 100, with the addition of 10 g/L substrate. The transformation was performed for 48 h at 30 °C and 200 rpm. Bar heights represent the averages of three biological replicates, and error bars represent standard deviation

Due to the use of the GAL promoter for expression of genes involved in the MVA pathway and the *AuoblA* gene, upon deletion of the *gal80* gene, the expression levels are regulated by glucose concentration. During the biomass accumulation phase in the whole-cell catalysis process, glucose is required as a carbon source. The target genes inside the cells will exhibit the highest expression levels only after the complete consumption of glucose [35]. Therefore, the timing of biomass collection is a critical factor. By setting the point when glucose in the culture medium is depleted (0 g/L) as a reference point (0 h), we investigated the efficiency of whole-cell conversion for producing OphF by collecting cells at 0 h, 4 h, 8 h and 12 h. As shown in Fig. 2A, after glucose depletion for 8 h, the highest catalytic efficiency was observed in the cell factory. Consequently, we tested the efficiency of the cell factory in utilizing different carbon sources (glucose, ethanol, acetate, pyruvate). After adding 10 g/L of carbon source to the transformation system and allowing sufficient reaction time, ethanol exhibited the highest yield, with an OphF production reaching 590 mg/L (Fig. 2B). The yield is lower when using glucose as a carbon source, possibly due to more catalytic steps from glucose to acetyl coenzyme A, a portion of carbon flows into bypass pathways, such as the synthesis of organic acids and glycerol. However, a shorter catalytic pathway does not necessarily result in a higher yield, as a carbon source, acetate showed the lowest yield, likely attributed to insufficient NADPH supply, as the synthesis of one molecule of OphF requires ten molecules of NADPH (Fig. 2C and E). Additionally, the consumption of acetate and pyruvate was accompanied by a rapid increase in the pH of the catalytic system, which is unfavorable for the stability of the reaction system (Additional file 1: Figure S3). Therefore, we opted for cost-effective ethanol, which exhibits a higher yield, as the preferred carbon source.

The pentose phosphate pathway (PPP) is identified as a crucial source of intracellular NADPH supply, engineering of this pathway are commonly employed strategies to enhance NADPH availability [41, 42]. However, when using ethanol as a substrate, the absence of the pentose phosphate pathway may lead to insufficient NADPH supply (Fig. 2C and E). POS5 was reported to mediate the ATP-driven conversion of NADH into NADPH in the yeast mitochondria [43]. Therefore, we overexpressed the endogenous POS5 in yeast and truncated the sequence located in the mitochondria, achieving the overexpression of tPOS5 in the cytoplasm. As shown in Fig. 2D, the strain Yoph18, overexpressing tPOS5, the titer of OphF increased to 649.6 mg/L. It also demonstrated that increasing the supply of NADPH can enhance the production of ophiobolins (Fig. 2C).

Acetyl-CoA is a crucial precursor in the mevalonate pathway, and the synthesis of one molecule of OphF requires 15 molecules of acetyl-CoA (Fig. 2E). Thus, we enhanced the metabolic pathway from ethanol to acetyl-CoA to elevate the supply of IPP/DMAPP. This strategy involved overexpressing the alcohol dehydrogenase gene *ADH2*, utilizing the glucose-regulatable promoter pGAL2. Subsequently, we constitutive overexpressed the NADP-dependent acetaldehyde dehydrogenase gene (*ALD6*) and the acetyl-CoA synthetase gene (*ACS1*) to obtain strain Yoph19, which resulted in a 14% increase in OphF production, reaching 742.3 mg/L (Fig. 2D).

**Fig. 3:**
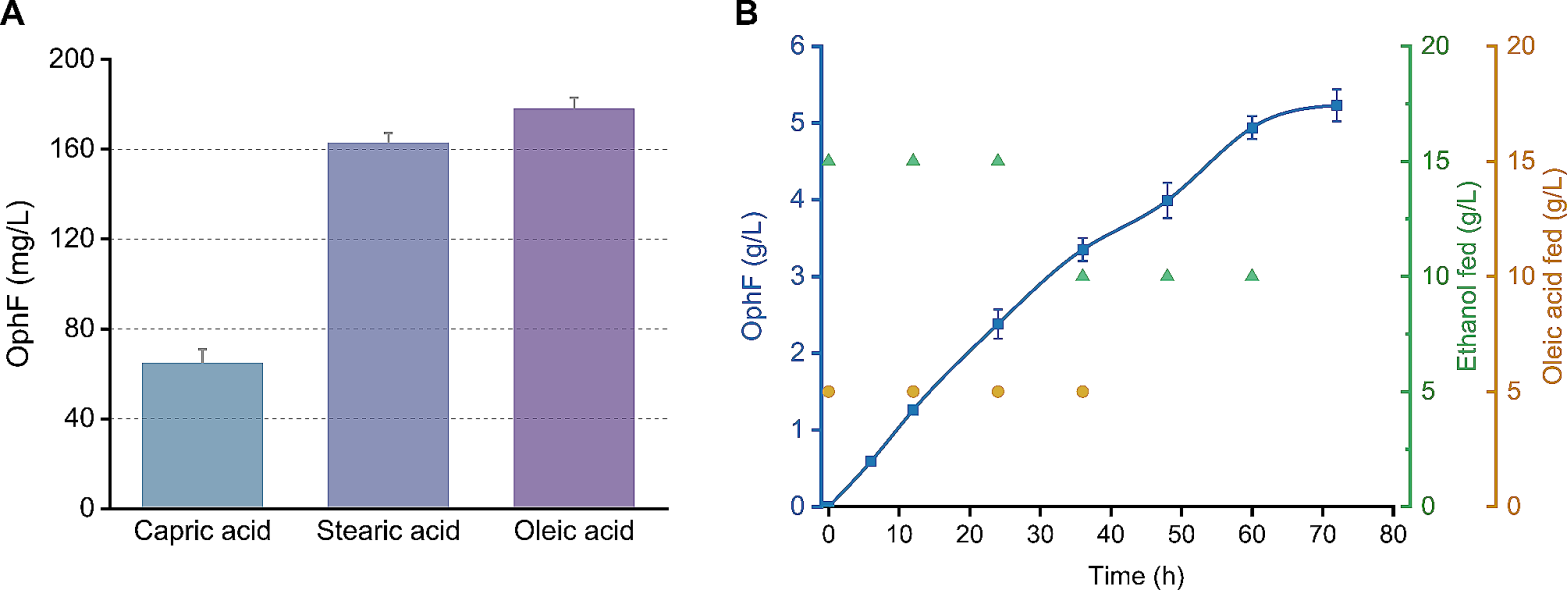
Utilizing ethanol and fatty acids for OphF synthesis. (**A**) The addition of long-chain fatty acids during whole-cell transformation is beneficial for OphF synthesis. Capric acid (C10) and stearic acid (C18), as well as the unsaturated fatty acid oleic acid (C18) were tested. (**B**) Fed-batch transformation for OphF production in 0.25 L shake-flasks. The green triangles and yellow dots represent the addition of ethanol and oleic acid, respectively. Bar heights represent the averages of three biological replicates, and error bars represent standard deviation

The β-oxidation of fatty acids also offers a substantial supply of acetyl-CoA, and yeast can uptake extracellular fatty acids as a carbon source [44, 45]. Therefore, we attempted to construct a strain capable of producing OphF by utilizing fatty acids. In *S. cerevisiae*, β-oxidation occurs exclusively within peroxisomes, and the peroxisomal membrane is impermeable to NAD(H) and acetyl-CoA [46, 47]. A strategy proven effective in increasing cytosolic acetyl-CoA levels involves anchoring carnitine acetyl-CoA transferase (Cat2) on the cytoplasmic side of peroxisomes [26]. Cat2 can transfer activated acetyl groups to carnitine, forming acetyl carnitine, which can be shuttled across membranes. Due to the bidirectionality of Cat2 catalysis, positioning cat2 on the surface of peroxisomes facilitates the direct conversion of acetyl carnitine into acetyl-CoA in the cytoplasm (Fig. 2E). We utilized the C-terminal 68 amino acids of Pex15 as the anchoring sequence [48] and constructed the Yoph20 strain with surface-localized cat2 on peroxisomes. Subsequently, we conducted fatty acid utilization experiments to synthesize OphF using different chain lengths of saturated fatty acids, capric acid (C10) and stearic acid (C18), as well as the unsaturated fatty acid oleic acid (C18). As shown in the Fig. 3A, long-chain fatty acids are more favorable for the synthesis of OphF. When oleic acid is used as the substrate, the yield of OphF reaches 178 mg/L. This result demonstrates that by adding fatty acids during the whole-cell conversion process, the β-oxidation pathway can serve as a supplement to intracellular acetyl-CoA supply. Subsequently, we conducted fed-batch whole-cell biotransformation using ethanol and oleic acid as carbon sources. By intermittently adding 75 g/L ethanol and 20 g/L oleic acid, the Yoph20 strain achieved a production of 5.1 g/L OphF after 72 h (Fig. 3B). This represents the highest reported sesterterpenoid yield to date, showing that whole-cell transformation is a practical approach for the industrial production of terpenoids.

We introduced Tween 80 as a cell-permeabilizing agent to facilitate the entry of substrates into cells and exiting ophiobolins from cells. This method has proven effective, but it may also compromise cell integrity and result in the leakage of cellular components, making it challenging to recycle cells [49]. Therefore, the transformation system still needs further optimization. For example, alternative chemical approaches could be explored, involving screening different detergents and solvents or employing physical methods, such as temperature shocks and ultrasonication, increasing the permeabilization levels of the cell wall and membrane. Generally, this approach allows the use of a broader range of substrates, facilitates the adjustment of transformation biomass, and does not require strict sterile conditions during the catalytic process, thereby reducing production costs. There are a few examples of using whole-cell transformation for de novo terpenoid compound synthesis; in our study, we have demonstrated the extraordinary value of this method.

### Selection of P450/CPR to oxidize ophiobolin F

**Fig. 4:**
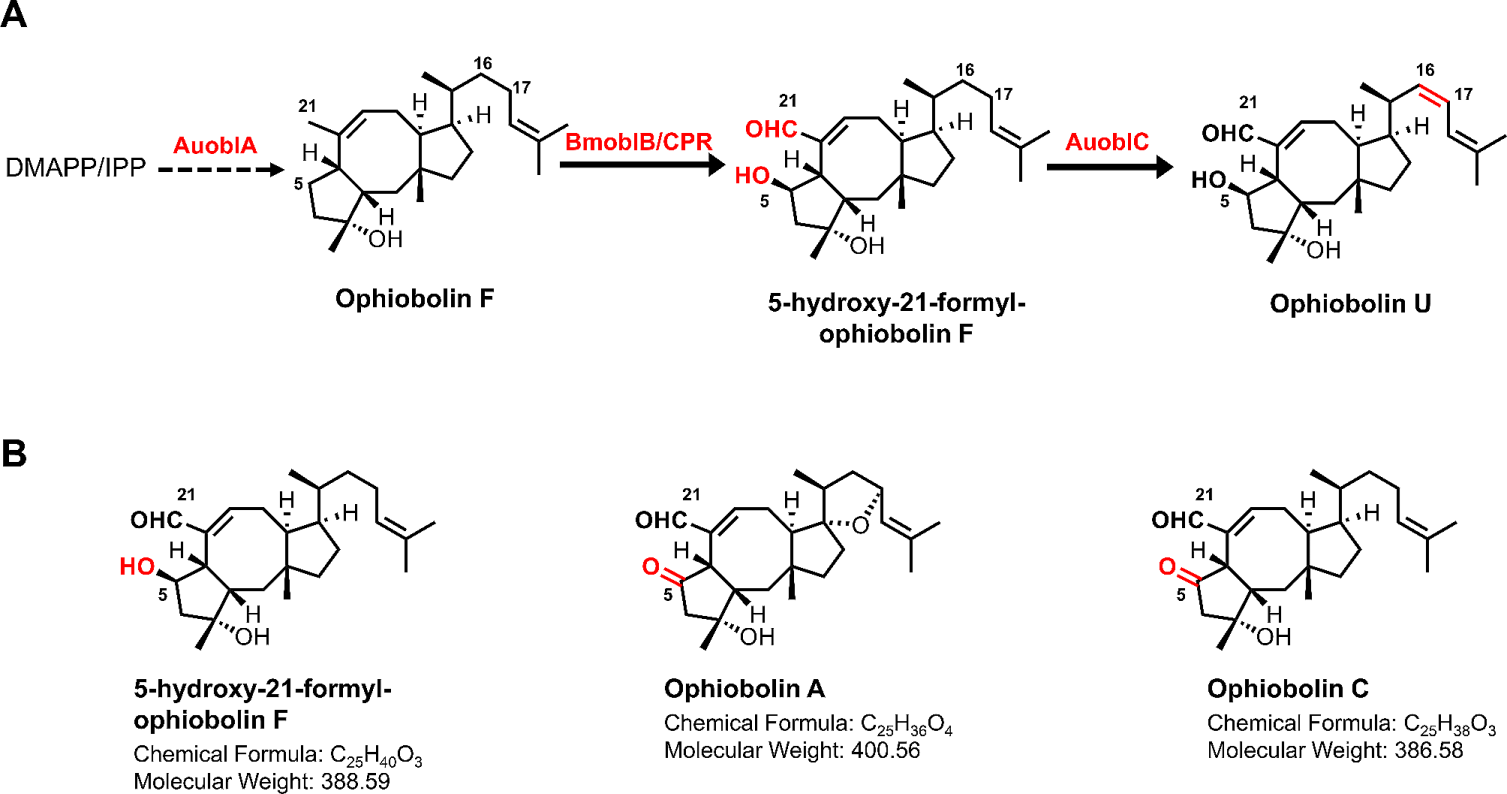
Scheme for biosynthesis pathway of 5-hydroxy-21-formyl-ophiobolin F and ophiobolin U in *S. cerevisiae*. AuoblA, ophiobolin F synthase, from *A. ustus* 094102; BmoblB, cytochrome P450 enzyme, from *Bipolaris maydis*; CPR, cytochrome P450 reductase; AuoblC, FAD-dependent oxidoreductase, from *A. ustus* 094102

Due to the lack of biological activity of OphF, we opted to perform oxidative modifications obtain bioactive products. The cytochrome P450 enzyme OblB catalyzes C5 and C21 oxidation of OphF, and the FAD-dependent oxidase OblC facilitates the C16-C17 dehydrogenation of the side chain [7], forming the final product (Fig. 4A). Because the activity of homologous enzymes from different species often varies, and their compatibility with the host may differ as well [12]. In order to screen for the most suitable P450 enzyme, we tested three P450 genes from different sources: *AuoblB* from *A. ustus* 094102 [7], *AcoblB* from *Aspergillus clavatus* [17], and *BmoblB* from *Bipolaris maydis* [50]. The strain Yoph33-35 was generated by expressing P450s in the Yoph14 strain. In previous studies, these P450 enzymes demonstrated the ability to oxidize OphF as a substrate, but the oxidation products obtained vary when the OblB genes were expressed in different hosts or sources [7, 17]. For instance, *A. ustus* 094102 primarily produces ophiobolin C, while *B. maydis* produces ophiobolin A. A common feature was the oxidation of the C5 position to a carbonyl group (Fig. 4B). Unexpectedly, in this study, all these P450 enzymes yielded the same primary product, 5-hydroxy-21-formyl-ophiobolin F (5-OH-21-CHO-ophF), a novel structure that has not been reported before (Fig. 4A, Additional file 1: Figure S4–S12, Table S4). This discrepancy suggests that the oxidation pathway of OphF may have not been fully elucidated, and the absence of specific unknown genes in *S. cerevisiae* may prevent the continuation of oxidation at the C5 position to form a carbonyl group. Another possibility is that the same strain can produce different ophiobolins under different cultivation conditions [51]. The mechanism behind this phenomenon requires further exploration. Subsequently, after catalysis by OblC (FAD-dependent oxidoreductase), ophiobolin U (OphU), which was initially discovered in *Aspergillus* sp. [52], was obtained (Fig. 4A, Additional file 1: Figure S5, Figure S13, S14, Table S4).

**Fig. 5:**
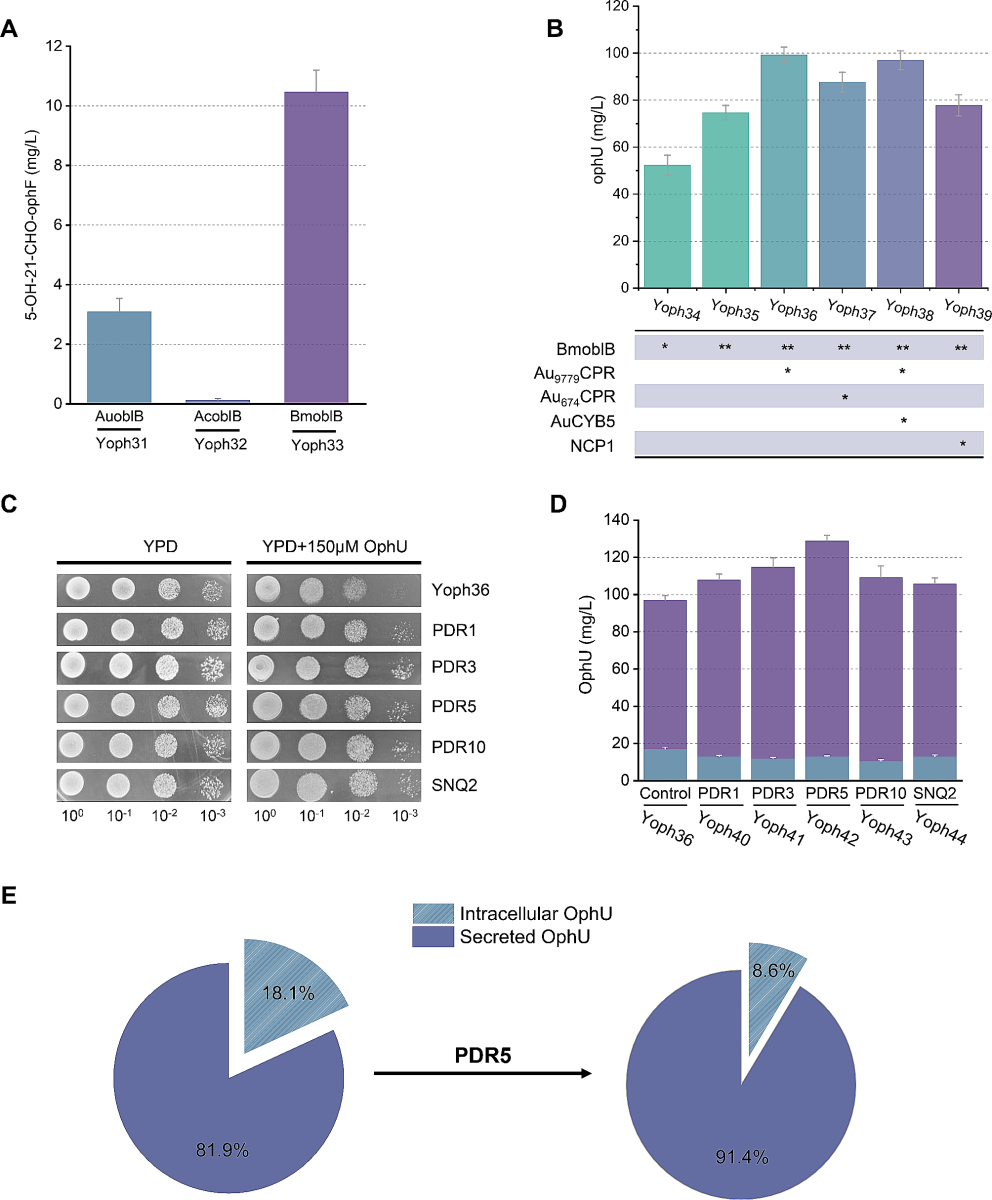
Improving OphU biosynthesis through P450-CPR optimization and PDR engineering. (**A**) Exploring the efficiency of P450 enzymes from different sources in the oxidation of OphF. (**B**) Improving OphU biosynthesis through optimization of *oblB* copy numbers and CPR screening. (**C**) Spot assays in Yoph36 and its PDR-engineered strains with 0 g/L and 150µmol/L OphU. The four spots from left to right represent different dilution factors (10^0^-10^− 3^). (**D**) Titer of OphU produced by PDR engineering strains. (**E**) Compared to the Yoph34 strain, in strains overexpressing PDR5, the efflux ratio of OphU increased from 81.9% to 91.4%

As shown in Fig. 5A, the *BmoblB* gene from *Bipolaris maydis* exhibited the highest catalytic efficiency, prompting us to select *BmoblB* for further investigation. Subsequently, we overexpressed FAD-dependent oxidoreductase *AuoblC* on the strain Yoph33 carrying *BmoblB*, yielding the strain Yoph34, which is capable of producing 53.3 mg/L of OphU.

An additional copy of *BmoblB* gene was integrated to obtain the strain Yoph35, resulting in a 40% increase in production for the compound OphU with double copies (Fig. 5B). However, the yield remained significantly lower than the accumulation of the precursor compound OphF. This suggests a substantial limitation on the catalysis of OphF by the cytochrome P450 enzyme BmoblB. BmoblB belongs to class II P450, and such P450 systems consist of the P450 enzyme and NADPH-cytochrome P450 reductase (CPR), which contains flavin adenine dinucleotide (FAD) and flavin mononucleotide (FMN) and both are membrane-bound proteins [53, 54]. Detection of OphU indicated that the endogenous CPR in *S. cerevisiae* (NCP1) could carry out the reaction. However, the lower product accumulation suggests that NCP1 might not be the optimal CPR for this process. Due to the crucial role of CYP-CPR interactions for optimal catalytic activity, some time the CYP-CPR pair of original strain is more efficient. Screening for introduced heterologous CPR is a common strategy [55, 56]. We sought to identify a more suitable CPR for OblB in the ophiobolin-producing strain, *A. ustus* 094102. Therefore, using Ncp1 as the query sequence, we identified two putative CPRs with high homology through BLASTp searches in the *A. ustus* 094102 genome. These putative CPRs, Au_674_CPR and Au_9779_CPR, returned 42% and 32.2% identities, respectively, to the amino acid sequence of NCP1.We utilized a constitutive promoter to express CPR, the overexpression of NCP1 resulted in only marginal improvement in product accumulation (Fig. 5B). In contrast, Au_9779_CPR as a CPR showed more favorable effects, with a 40% increase in yield, emphasizing the importance of optimizing and selecting the CPR (Fig. 5B). Additionally, cytochrome b5 (CYB5) has been identified to enhance the electron transfer efficiency of P450 enzymes [11]. Therefore, we introduced a putative CYB5 (AuCYB5) from *A. ustus* 094102, but it did not positively impact product accumulation (Fig. 5B).

### Promoting product efflux to enhance ophiobolin U production

Due to the significantly slowed growth of the engineered strain overexpressing the BmoblB, we hypothesized that the catalytic products of the P450 enzyme might exert a toxic effect on the cells (Additional file 1: Figure S2). Although a majority of OphU is extruded to the extracellular environment during fermentation, approximately 18% still accumulates intracellularly (Fig. 5E). As evident from the results of the spot dilution growth assay (Fig. 5C), growth inhibition can be observed in YPD medium containing 150 µM OphU, and the inhibitory effect of OphU is significantly stronger than OphF (Additional file 1: Figure S15).

In the biosynthetic gene cluster of ophiobolins in *A. ustus* 094102, the ATP-binding cassette (ABC) transporter protein OblD facilitates the transport of ophiobolins, thereby alleviating inhibition on cell growth. In the native strain producing ophiobolins, the gene encoding the transporter protein, AuoblD, exhibited a higher expression level (increased by 5.6-fold) under the oph^+^ condition than the oph^−^ condition, the absence of OblD significantly impacts the synthesis of products catalyzed by P450 enzymes [7]. ABC transporters play a crucial role in mediating pleiotropic drug resistance (PDR) in microorganisms against various stress conditions [57]. Due to the failure to synthesize the *OblD* gene, we could not express the codon-optimized OblD in *S. cerevisiae*. And we expressed the non-codon-optimized AuoblD from *A. ustus* 094102, however, we did not observe an enhancement in the production of ophU (data not shown).

As an alternative approach, we attempted to enhance product efflux and increase yield by screening PDR genes in the *S. cerevisiae* genome. Using AuoblD as a query sequence, we conducted a BLASTp search on the *S. cerevisiae* genome, identifying three ABC transporter proteins (PDR5, PDR10, SNQ2) with high homology. Considering the transcriptional regulation of these genes by PDR1/3 activation [58], *PDR1, PDR3, PDR5, PDR10*, and *SNQ2* genes were integrated into strain Yoph36, respectively, and engineered strains Yoph40, Yoph41, Yoph42, Yoph43 and Yoph44 were constructed. As observed in in Fig. 5C, although the expression of these genes did not completely relieve the growth inhibition, there was varying degrees of improvement compared to the control strain (Yoph36). The most significantly enhanced OphU production was observed in strain Yoph42, overexpresses PDR5, with a production of OphU reaching 128.9 mg/L (Fig. 5D). Moreover, the proportion of OphU excreted to the extracellular increased from 81.9% to 91.4% (Fig. 5E). This result demonstrates that PDR engineering is a promising approach for the toxic compounds biosynthesis. In subsequent studies, PDR engineering could also improve the synthesis of 5-OH-21-CHO-OphF, which possesses higher medicinal potential (Table 2).

Whole-cell transformation can efficiently produce the ophiobolin skeleton compound OphF. Unfortunately, the strains (Yoph33 and Yoph42) producing OphF oxidation products did not show an increased titer through whole-cell transformation, and the reasons for this are unknown. Additionally, we attempted to directly use OphF as a substrate in feeding experiments with strain expressing OblB. However, no detectable oxidation products were observed, suggesting that extracellular OphF is difficult to transport into the cells.

### The cytotoxicity testing of OphU and 5-OH-21-CHO-OphF

After obtaining the oxidized ophiobolins, we tested the biological activity of OphU and 5-OH-21-CHO-OphF on several cancer cell lines. The novel molecule 5-OH-21-CHO-OphF showed a strong cytotoxic activity against A2780 (human ovarian cancer) cell line with IC_50_ values of 1.33 µM, Siha (human cervical carcinoma) cell line with IC_50_ values of 4.64 µM and MDA-MB-231 (human TNBC) cell line with IC_50_ values of 10.71 µM, respectively. OphU showed a moderate cytotoxicity against A2780 cell lines, with an IC_50_ value of 11.73 µM (Table 2). Compared to OphU, 5-OH-21-CHO-OphF exhibits superior anti-tumor activities, suggesting that the oxidation reaction at C16-C17 catalyzed by OblC is not a critical factor in generating biological activity. Comparing the studies reported on OphA, OphC, and OphK, it is apparent that oxidation of the C-5 position to a hydroxyl or carbonyl group does not significantly affect their cytotoxic activity. Though the hydroxyl group at position C-3 and the aldehyde group at position C-21 are essential for the biological activity of ophiobolins [1, 7]. However, considering the strains produced significantly more OphU than 5-OH-21-CHO-OphF, 5-OH-21-CHO-OphF may exert more significant toxicity to the cells, and the presence of OblC may function, in a sense, as an “antidote” for ophiobolins-producing strains. This phenomenon has not been found in other studies, may be a unique phenomenon observed only in yeast.

**Table 2:** The IC_50_ values (µM) of OphF, OphU and 5-OH-21-CHO-OphF on A2780, Siha, and MDA-MB-231 cell lines

	A2780	Siha	MDA-MB-231
Ophiobolin F	> 40	> 40	> 40
Ophiobolin U	11.73 ± 0.63	40.23 ± 1.37	> 40
5-OH-21-CHO-OphF	1.33 ± 0.54	4.64 ± 0.27	10.71 ± 0.43
Adriamycin	1.01 ± 0.13	N/A	0.42 ± 0.11
Cisplatin	N/A	2.79 ± 0.27	N/A

Adriamycin and cisplatin used as positive control

## Conclusion

This study established efficient cell factories based on *S. cerevisiae*, enabling de novo biosynthesis of the ophiobolin skeleton OphF and oxidized ophiobolins derivatives. By overexpressing ophiobolin biosynthetic genes and precursor supply pathway, the yield of OphF exceeded 5 g/L using whole-cell transformation and is currently the highest yield in *S. cerevisiae* for the biosynthesis of sesterterpenoids. By expressing P450 enzymes for post-modification of OphF, we obtained a novel structure with potent cytotoxicity, 5-OH-21-CHO-OphF. We also developed a strain with high OphU productivity by optimizing CPR and PDR genes. Our work provides a paradigm for synthesizing value-added sesterterpenoids with complex structures.

### Electronic supplementary material

Below is the link to the electronic supplementary material.

Supplementary Material 1

## Data Availability

No datasets were generated or analysed during the current study.

## References

[1] Tian W, Deng Z, Hong K (2017). The biological activities of sesterterpenoid-type ophiobolins. Mar Drugs.

[2] Wen H, Zhong Y, Yin Y, Qin K, Yang L, Li D (2022). A marine-derived small molecule induces immunogenic cell death against triple-negative breast cancer through ER stress-CHOP pathway. Int J Biol Sci.

[3] Rowley M, Tsukamoto M, Kishi Y (1989). Total synthesis of (+)-ophiobolin C. J Am Chem Soc.

[4] Thach DQ, Brill ZG, Grover HK, Esguerra KV, Thompson JK, Maimone TJ (2020). Total synthesis of (+)-6-epi‐ophiobolin A. Angew Chem Int Ed.

[5] Brill ZG, Grover HK, Maimone TJ (2016). Enantioselective synthesis of an ophiobolin sesterterpene via a programmed radical cascade. Science.

[6] Tsuna K, Noguchi N, Nakada M (2011). Convergent total synthesis of (+)-ophiobolin A. Angew Chem Int Ed.

[7] Yan J, Pang J, Liang J, Yu W, Liao X, Aobulikasimu A (2022). The biosynthesis and transport of ophiobolins in *aspergillus ustus* 094102. Int J Mol Sci.

[8] Liu Z, Huang M, Chen H, Lu X, Tian Y, Hu P (2024). Metabolic engineering of *Yarrowia Lipolytica* for high-level production of squalene. Bioresour Technol.

[9] Ma Y, Liu N, Greisen P, Li J, Qiao K, Huang S (2022). Removal of lycopene substrate inhibition enables high carotenoid productivity in *Yarrowia Lipolytica*. Nat Commun.

[10] Ye Z, Shi B, Huang Y, Ma T, Xiang Z, Hu B (2022). Revolution of vitamin E production by starting from microbial fermented farnesene to isophytol. Innovation.

[11] Paddon CJ, Westfall PJ, Pitera DJ, Benjamin K, Fisher K, McPhee D (2013). High-level semi-synthetic production of the potent antimalarial artemisinin. Nature.

[12] Zhang J, Hansen LG, Gudich O, Viehrig K, Lassen LMM, Schrübbers L (2022). A microbial supply chain for production of the anti-cancer drug vinblastine. Nature.

[13] Gao J, Zuo Y, Xiao F, Wang Y, Li D, Xu J (2023). Biosynthesis of catharanthine in engineered *Pichia pastoris*. Nat Synthesis.

[14] Kong X, Wu Y, Yu W, Liu Y, Li J, Du G (2023). Efficient synthesis of limonene in *Saccharomyces cerevisiae* using combinatorial metabolic engineering strategies. J Agric Food Chem.

[15] Chai H, Yin R, Liu Y, Meng H, Zhou X, Zhou G (2016). Sesterterpene ophiobolin biosynthesis involving multiple gene clusters in *aspergillus ustus*. Sci Rep.

[16] Chiba R, Minami A, Gomi K, Oikawa H (2013). Identification of ophiobolin F synthase by a genome mining approach: a sesterterpene synthase from *Aspergillus Clavatus*. Org Lett.

[17] Narita K, Chiba R, Minami A, Kodama M, Fujii I, Gomi K (2016). Multiple oxidative modifications in the ophiobolin biosynthesis: P450 oxidations found in genome mining. Org Lett.

[18] Yuan W, Lv S, Chen L, Zhao Y, Deng Z, Hong K (2019). Production of sesterterpene ophiobolin by a bifunctional terpene synthase in *Escherichia coli*. Appl Microbiol Biotechnol.

[19] Mai W, Hong K (2019). Heterologous expression of a fungal cytochrome P450 in *Escherichia coli*. Microbiol China.

[20] Arendt P, Miettinen K, Pollier J, De Rycke R, Callewaert N, GoossensM A (2017). An endoplasmic reticulum-engineered yeast platform for overproduction of triterpenoids. Metab Eng.

[21] Yee DA, DeNicola AB, Billingsley JM, Creso JG, Subrahmanyam V, Tang Y (2019). Engineered mitochondrial production of monoterpenes in *Saccharomyces cerevisiae*. Metab Eng.

[22] Chatzivasileiou AO, Ward V, Edgar SM, Stephanopoulos G (2019). Two-step pathway for isoprenoid synthesis. Proc Natl Acad Sci U S A.

[23] Siemon T, Wang Z, Bian G, Seitz T, Ye Z, Lu Y (2020). Semisynthesis of plant-derived englerin a enabled by microbe engineering of Guaia-6,10(14)-diene as building block. J Am Chem Soc.

[24] Cao X, Yu W, Chen Y, Yang S, Zhao ZK, Nielsen J (2023). Engineering yeast for high-level production of diterpenoid sclareol. Metab Eng.

[25] Chen Y, Daviet L, Schalk M, Siewers V, Nielsen J (2013). Establishing a platform cell factory through engineering of yeast acetyl-CoA metabolism. Metab Eng.

[26] Yocum H, Bassett S, Silva NA, Da (2022). Enhanced production of acetyl-CoA based products via peroxisomal surface display in *Saccharomyces cerevisiae*. Proc Natl Acad Sci U S A.

[27] Kwak S, Yun EJ, Lane S, Oh EJ, Kim KH, Jin Y (2019). Redirection of the glycolytic flux enhances isoprenoid production in *Saccharomyces cerevisiae*. Biotechnol J.

[28] Verho R, Londesborough J, Penttilä M, Richard P (2003). Engineering Redox cofactor regeneration for improved pentose fermentation in *Saccharomyces cerevisiae*. Appl Environ Microbiol.

[29] Lee SY, Kim HU (2015). Systems strategies for developing industrial microbial strains. Nat Biotechnol.

[30] Lin B, Tao Y (2017). Whole-cell biocatalysts by design. Microb Cell Fact.

[31] Niu F-X, He X, Wu YQ, Liu JZ (2018). Enhancing production of pinene in *Escherichia coli* by using a combination of tolerance, evolution, and modular co-culture engineering. Front Microbiol.

[32] Naito Y, Hino K, Bono H, Ui-Tei K (2015). CRISPRdirect: software for designing CRISPR/Cas guide RNA with reduced off-target sites. Bioinformatics.

[33] Zhang Y, Wang J, Wang Z, Zhang Y, Shi S, Nielsen J (2019). A gRNA-tRNA array for CRISPR-Cas9 based rapid multiplexed genome editing in *Saccharomyces cerevisiae*. Nat Commun.

[34] Gietz RD, Schiestl RH (2007). High-efficiency yeast transformation using the LiAc/SS carrier DNA/PEG method. Nat Protoc.

[35] Ricci-Tam C, Ben-Zion I, Wang J, Palme J, Li A, Savir Y (2021). Decoupling transcription factor expression and activity enables dimmer switch gene regulation. Science.

[36] Ma B, Liu M, Li ZH, Tao X, Wei DZ, Wang FQ (2019). Significantly enhanced production of patchoulol in metabolically engineered *Saccharomyces cerevisiae*. J Agric Food Chem.

[37] Faulkner A, Chen X, Rush J, Horazdovsky B, Waechter CJ, Carman GM (1998). The lpp1 and dpp1 gene products account for most of the isoprenoid phosphate phosphatase activities in *Saccharomyces cerevisiae*. J Biol Chem.

[38] Zhang C, Liu J, Zhao F, Lu C, Zhao GR, Lu W (2018). Production of sesquiterpenoid zerumbone from metabolic engineered *Saccharomyces cerevisiae*. Metab Eng.

[39] Trikka FA, Nikolaidis A, Athanasakoglou A, Andreadelli A, Ignea C, Kotta K (2015). Iterative carotenogenic screens identify combinations of yeast gene deletions that enhance sclareol production. Microb Cell Fact.

[40] Chen Y, Wang Y, Liu M, Qu J, Yao M, Li B (2019). Primary and secondary metabolic effects of a key gene deletion (∆*YPL062W*) in metabolically engineered terpenoid-producing *Saccharomyces cerevisiae*. Appl Environ Microbiol.

[41] Chen R, Gao J, Yu W, Chen X, Zhai X, Chen Y (2022). Engineering cofactor supply and recycling to drive phenolic acid biosynthesis in yeast. Nat Chem Biol.

[42] Yang J, Liang J, Shao L, Liu L, Gao K, Zhang JL (2020). Green production of silybin and isosilybin by merging metabolic engineering approaches and enzymatic catalysis. Metab Eng.

[43] Hou J, Lages NF, Oldiges M, Vemuri GN (2009). Metabolic impact of redox cofactor perturbations in *Saccharomyces cerevisiae*. Metab Eng.

[44] Hiltunen JK, Mursula AM, Rottensteiner H, Wierenga RK, Kastaniotis AJ, Gurvitz A (2003). The biochemistry of peroxisomal β-oxidation in the yeast *Saccharomyces cerevisiae*. FEMS Microbiol Rev.

[45] Sun Y, Sun L, Shang F, Yan G (2016). Enhanced production of β-carotene in recombinant *Saccharomyces cerevisiae* by inverse metabolic engineering with supplementation of unsaturated fatty acids. Process Biochem.

[46] Kunau WH, Dommes V, Schulzt H (1995). β-oxidation of fatty acids in mitochondria, peroxisomes, and bacteria: a century of continued progress. Prog Lipid Res.

[47] Van Roermund CWT, Elgersma Y, Singh N, Wanders RJA, Tabak HF (1995). The membrane of peroxisomes in *Saccharomyces cerevisiae* is impermeable to NAD(H) and acetyl-CoA under in vivo conditions. EMBO J.

[48] Halbach A, Landgraf C, Lorenzen S, Rosenkranz K, Volkmer-Engert R, Erdmann R (2006). Targeting of the tail-anchored peroxisomal membrane proteins PEX26 and PEX15 occurs through C-terminal PEX19-binding sites. J Cell Sci.

[49] de Carvalho CCCR (2017). Whole cell biocatalysts: essential workers from Nature to the industry. Microb Biotechnol.

[50] Sugawara F, Strobel G, Strange RN, Siedow JN, Van Duyne GD, Clardy J (1987). Phytotoxins from the pathogenic fungi *Drechslera maydis* and *Drechslera sorghicola*. Proc Natl Acad Sci U S A.

[51] Li E, Clark AM, Rotella DP, Hufford CD. Microbial metabolites of ophiobolin a and antimicrobial evaluation of ophiobolins. J Nat Prod. 1995; 58, (1). 10.1021/np50115a009 7760080

[52] Bladt TT, Durr C, Knudsen PB, Kildgaard S, Frisvad JC, Gotfredsen CH (2013). Bio-activity and dereplication-based discovery of ophiobolins and other fungal secondary metabolites targeting leukemia cells. Molecules.

[53] Urlacher VB, Girhard M (2019). Cytochrome P450 monooxygenases in biotechnology and synthetic biology. Trends Biotechnol.

[54] Bernhardt R, Urlacher VB (2014). Cytochromes P450 as promising catalysts for biotechnological application: chances and limitations. Appl Microbiol Biotechnol.

[55] Gold ND, Fossati E, Hansen CC, DIfalco M, Douchin V, Martin VJJ (2018). A combinatorial approach to study cytochrome P450 enzymes for de novo production of steviol glucosides in baker’s yeast. ACS Synth Biol.

[56] Milne N, Thomsen P, Mølgaard Knudsen N, Rubaszka P, Kristensen M, Borodina I (2020). Metabolic engineering of *Saccharomyces cerevisiae* for the de novo production of psilocybin and related tryptamine derivatives. Metab Eng.

[57] Wolfger H, Mamnun YM, Kuchler K (2001). Fungal ABC proteins: pleiotropic drug resistance, stress response and cellular detoxification. Res Microbiol.

[58] Jungwirth H, Kuchler K (2006). Yeast ABC transporters-A tale of sex, stress, drugs and aging. FEBS Lett.

