# Supplementary material for: De novo production of bioactive sesterterpenoid ophiobolins in Saccharomyces cerevisiae cell factories
Source: Microb Cell Fact. 2024 May 6;23:129. doi: 10.1186/s12934-024-02406-0 (PMC11071210; doi:10.1186/s12934-024-02406-0)
Supplement: Supplementary file 1 — Supplementary Material 1 [file 12934_2024_2406_MOESM1_ESM.docx]

**Figure S1**. Flowchart of engineered strains construction.


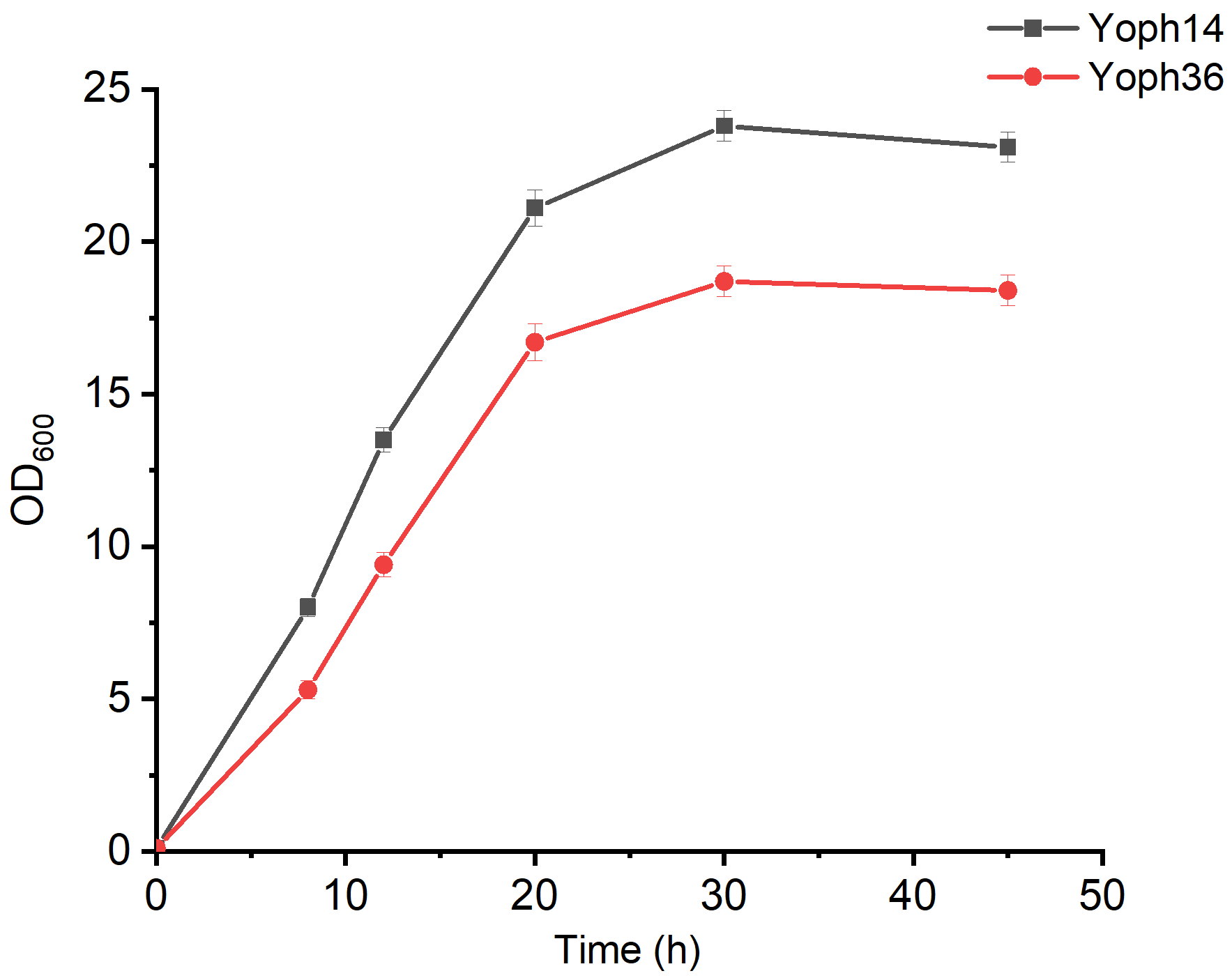


**Figure S2.** The growth curves of Yoph14 and Yoph36 strains.


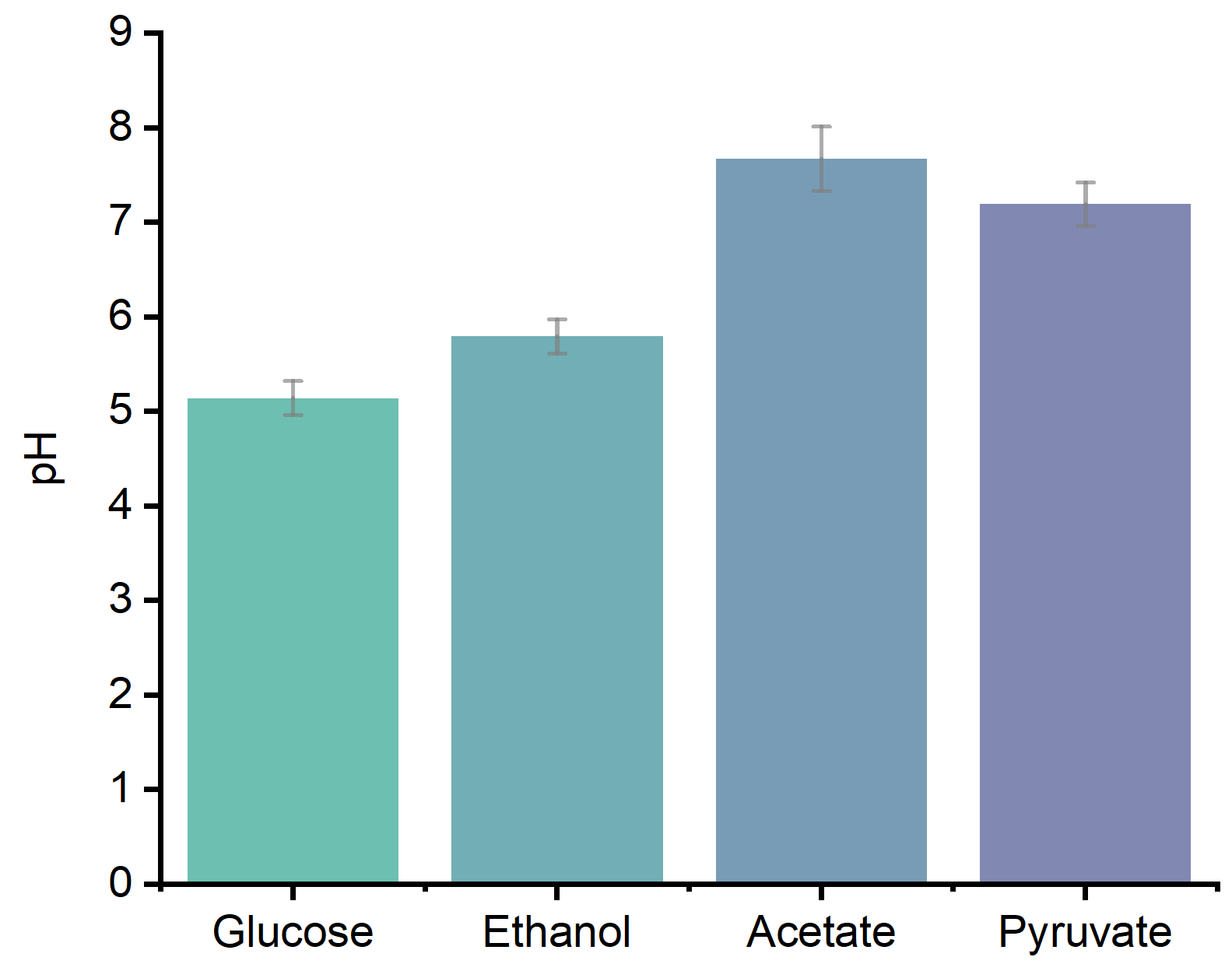


**Figure S3.** The pH variation with different carbon sources in whole-cell transformation.

Add 10 g/L of each carbon source, with an initial pH of 6.0. Measure the pH again after 6 hours of shaking flask reaction.


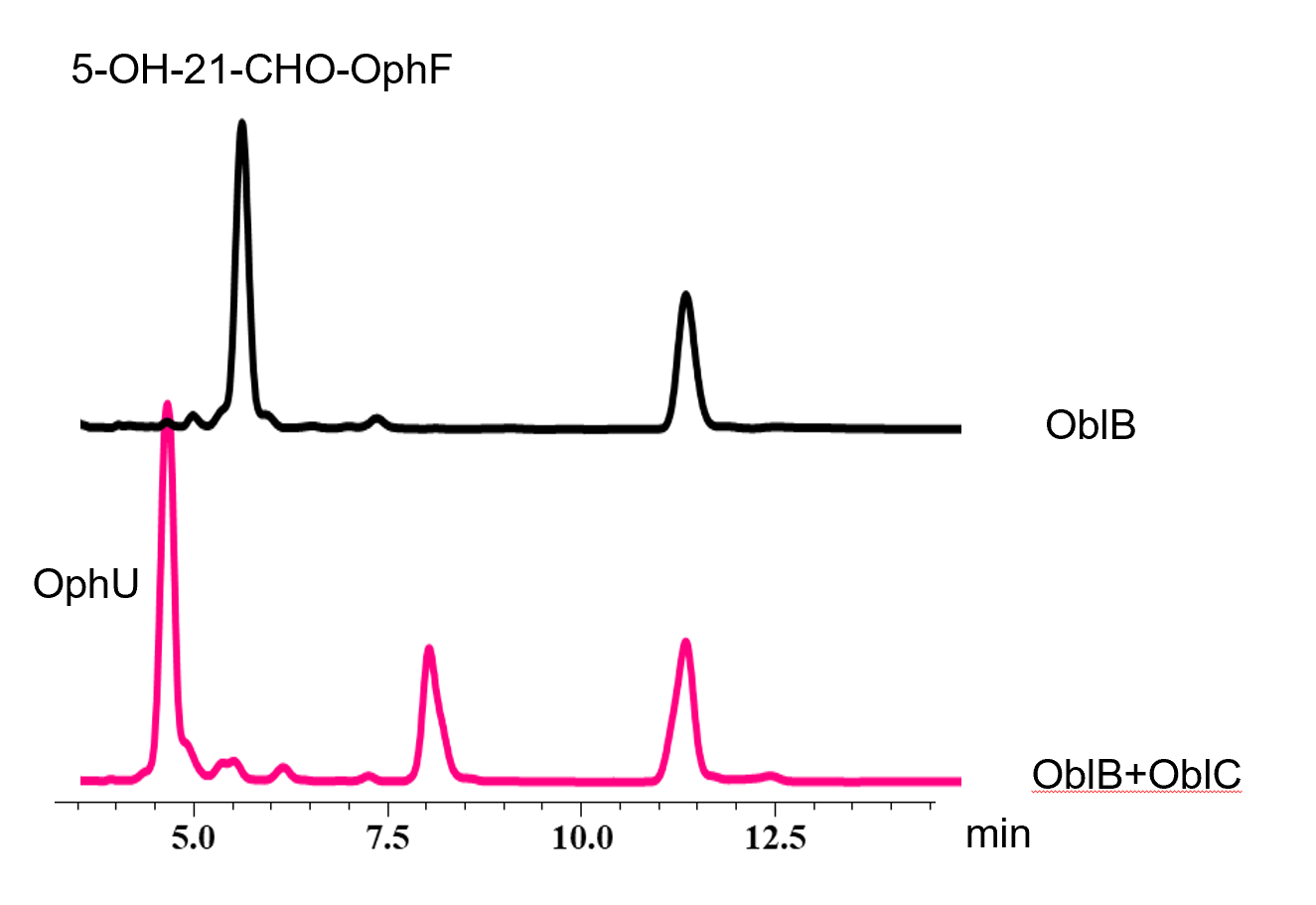


**Figure S4**. HPLC detection of 5-OH-21-CHO-OphF and OphU at 240 nm.


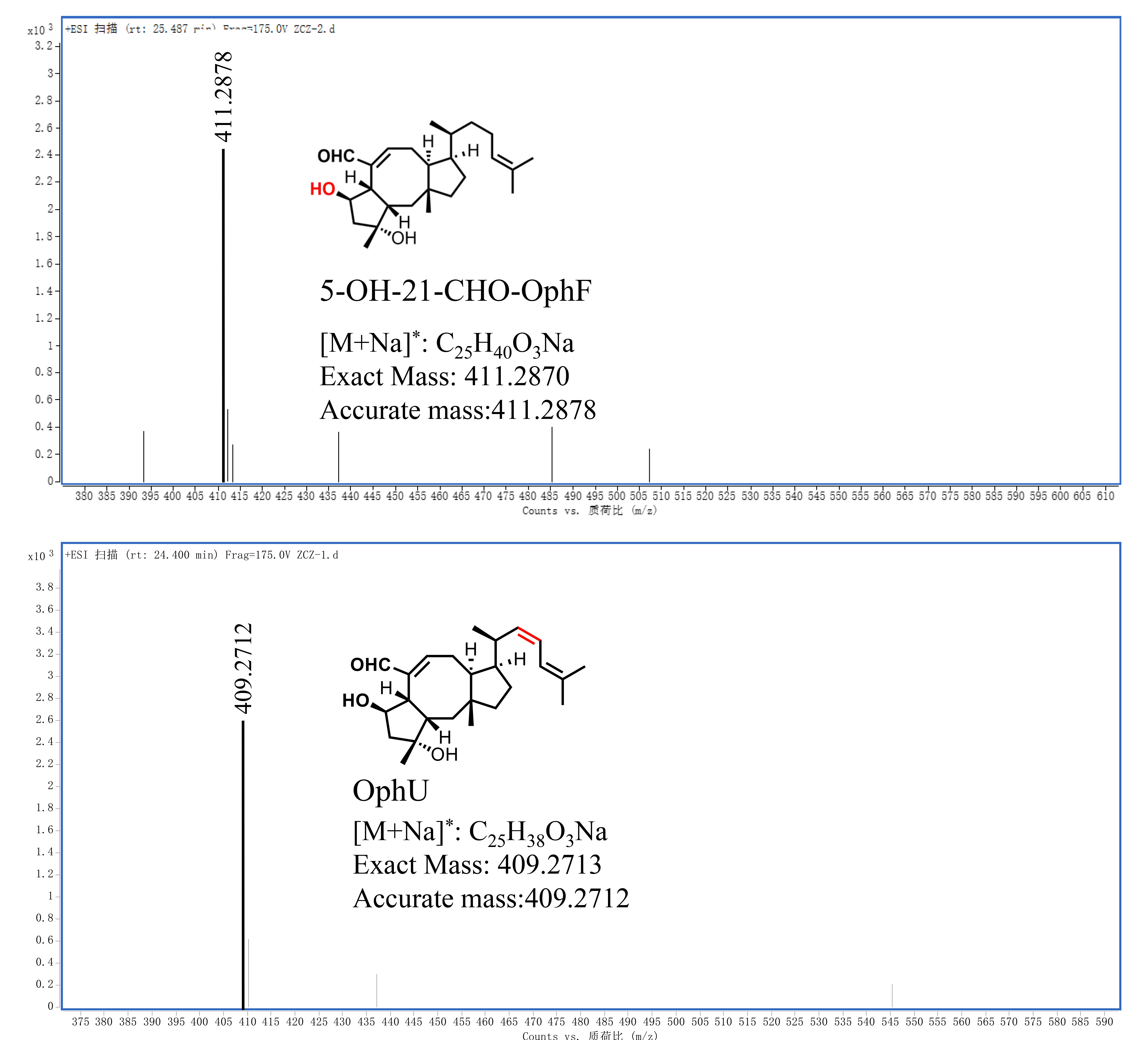


**Figure S5.** LC-MS analysis of the 5-OH-21-CHO-OphF and OphU.

**Figure S6.** HMBC, COSY and NOESY correlations of 5-OH-21-CHO-OphF (600 MHz, CDCl_3_).

**Figure S7.** ^1^H NMR spectrum of 5-OH-21-CHO-OphF (600 MHz, CDCl_3_).

**Figure S8.** ^13^C NMR spectrum of 5-OH-21-CHO-OphF (150 MHz, CDCl_3_).

**Figure S9.** HSQC spectrum of 5-OH-21-CHO-OphF (600 MHz, CDCl_3_).

**Figure S10.** HMBC spectrum of 5-OH-21-CHO-OphF (600 MHz, CDCl_3_).

**Figure S11.** NOESY spectrum of 5-OH-21-CHO-OphF (600 MHz, CDCl_3_).


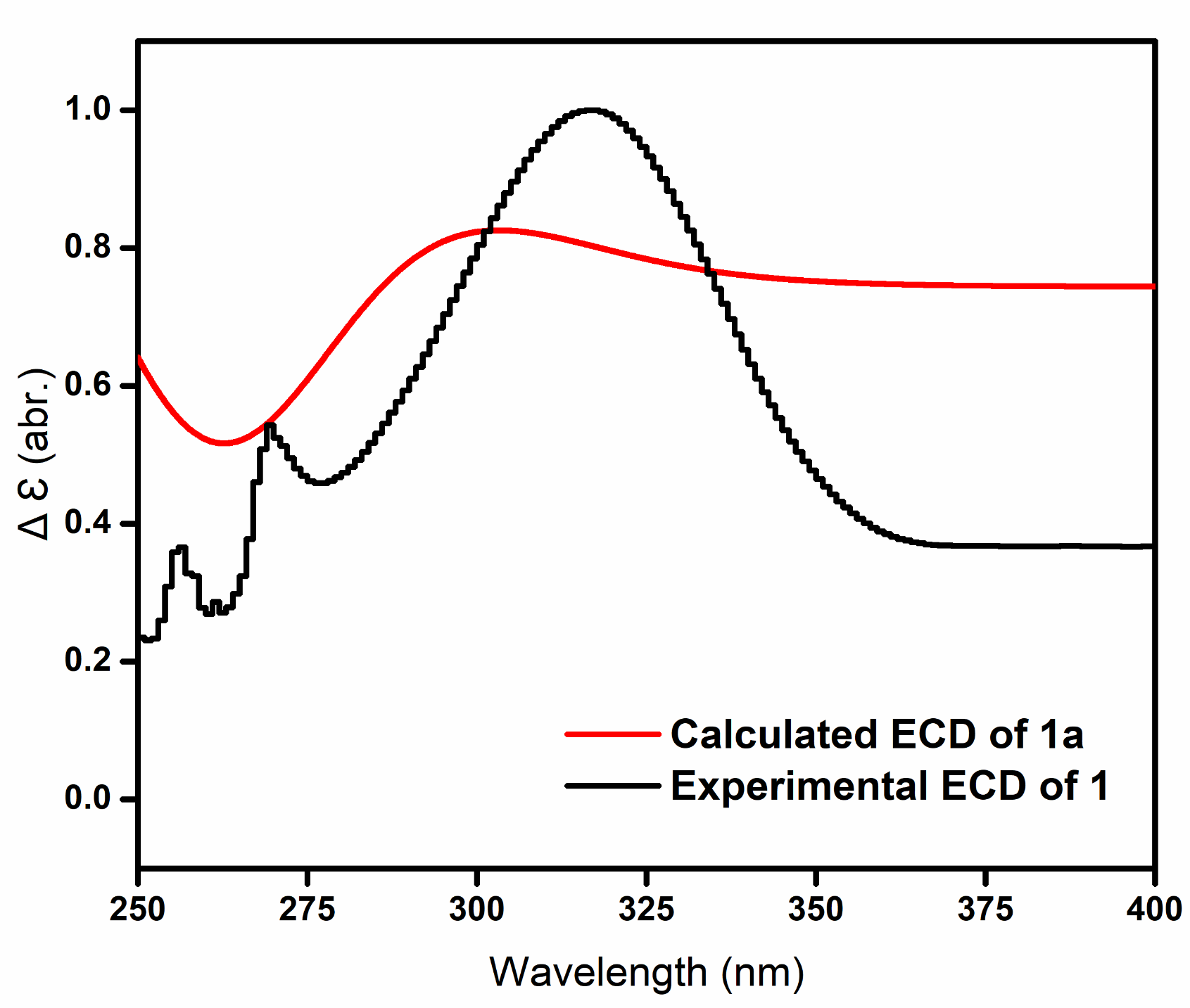
**Figure S12.** ECD spectra of 5-OH-21-CHO-OphF.


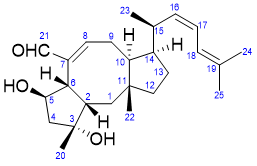


**Figure S13.** ^1^H NMR spectrum of OphU (600 MHz, CDCl_3_).


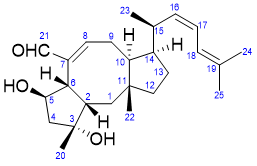


**Figure S14.** ^13^C NMR spectrum of OphU (150 MHz, CDCl_3_).


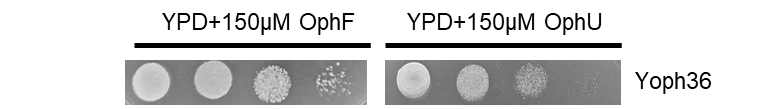


**Figure S15**. Spot assays in Yoph36 with 150μmol/L OphF and OphU.

**Structure elucidation of ophiobolins**

5-OH-21-CHO-OphF (**1)** was obtained as colorless oil. The molecular formula of **1** was determined to be C_25_H_40_O_3_ provided 6 degrees of unsaturation on the basis of ESI-HRMS peak at m/z 411.2878 (calcd for C_25_H_40_O_3_Na 411.2875). The ^1^H NMR spectrum (**Table S4**) of **1** showed five methyl groups [*δ*_H_ 1.71 (s, Me-25), 1.60 (s, Me-24), 1.28 (s, Me-19), 0.94 (s, Me-21), 0.79 (d, *J* = 6.82 Hz, Me-22)], two olefinic protons [*δ*_H_ 7.01 (m, H-8), 5.10 (dddd, *J* = 9.94, 7.08, 2.74, 1.32 Hz, H-18)], and one aldehyde proton [*δ*_H_ 9.26 (d, *J* = 0.96 Hz, H-21)]. The ^13^C NMR data combined with the HSQC spectrum provided 25 signals displayed as follows: five methyls, eight methylenes, eight methines including two sp^2^ ones, and four quaternary carbons including one carbonyl (*δ*_C_ 198.29). The analysis of the ^1^H-^1^H COSY spectrum (**Figure S5**) provided two isolated proton spin systems displayed as C-1‒C-2‒C-6‒C-5‒C-4 and C-8‒C-9‒C-10‒C-14(C-13‒C12) ‒C-15(C-23) ‒C-16‒C-17‒C-18. These data were similar to that of ophiobolin U (**2**) except the absence of the olefinic double bone from C-16 to C-17. The COSY correlations of H-15/H-16 (*δ*_H_ 1.15, 1.23)/H-17 (*δ*_H_ 1.66, 1.97)/H-18 as well as the key HMBC correlations from H-23 (*δ*_H_ 0.79) to C-16 (*δ*_C_ 37.2), H-15 (*δ*_H_ 1.65) to C-16 and C-17 (*δ*_C_ 26.6), H-18 (*δ*_H_ 5.10) to C-16 and C-17 supported the structure of **1**. The same relative configuration as **2** was established by the NOESY correlations between H-2 (*δ*_H_ 2.31) and H-4a (*δ*_H_ 1.87), H-4a and H-6 (*δ*_H_ 3.02), H-2 and H-22 (*δ*_H_ 0.94), H-22 and H-13a (*δ*_H_ 1.42), H-13a and H-23 (*δ*_H_ 0.79), indicating these protons on the same side. Meanwhile, the strong NOESY correlations between H-5 and H-4b (*δ*_H_ 2.68) locating these protons on the other side. To confirm the absolute configuration of **1**, the stereoisomer **1a** (2S, 3R, 5R, 6S, 10S, 11R, 14R, 15S), was calculated weighted ECD spectrum with time-dependent density functional theory (TDDFT) at the B3LYP/6-311G (2d, p) level. The experiment ECD spectrum of **1** was consistent with **1a**, indicating the (2S, 3R, 5R, 6S, 10S, 11R, 14R, 15S) configuration.

**Table S1**. Primers used in this study.

| Primer purpose and name | Sequence (5’–3’) |
| --- | --- |
| Construct for knocking out the *gal80* gene | |
| Oph-1-gal80 | ATTGGGTGCCTCTATGATGG |
| Oph-2-gal80 | GATGCTTTGCGAATTCGCGACGGGAGTGGAAAGAAC |
| Oph-3-gal80 | TCGCGAATTCGCAAAGCATCTTGCCCTGTGC |
| Oph-4-gal80 | AGCGCAATTTACTAATGGCAT |
| Construct for P_GAL10_-AuoblA-T_ADH1_ with ChrI-1 homologous arm | |
| Oph-5- ChrI | AAACAGCTATGACCATGATACTAGTAACTTGCTCCCTTGAAAGCG |
| Oph-6- ChrI | TAACCTGGCCTTTGGTCGTTGCATTGACACACATCTCAAG |
| Oph-7- ChrI | ATGAGGTCGCTCAGGAATTACACGGCAGAAAGATTTTCGCTAC |
| Oph-8- ChrI | TTAACTATGCGGCATCACTAGTCCAATGATGGAGGTTCTTCTC |
| Oph-9-Pgal10 | AACGACCAAAGGCCAGGTTACTGCCAATTTTTC |
| Oph-10-Pgal10 | GAATTCAATTCGAATTTTCAAAAATTCTTAC |
| Oph-11-AuoblA | AATTTTTGAAAATTCGAATTGAATTCATGGAATACAAGTACTCC |
| Oph-12-AuoblA | GCGAAGAATTGTTAATTAAGGAGCTCTTAAACTTTCAACAATTC |
| Oph-13-Tadh1 | TGTAATTCCTGAGCGACCTCATGCTATACC |
| Oph-14-Tadh1 | GAGCTCCTTAATTAACAATTCTTC |
| Construct for T_HMG1_-*tHMG1*-P_GAL10_-P_GL1_-*ERG13-T_ERG13_* with ChrIII-1 homologous arm | |
| Oph-15-Pgal1/10 | AGTGAGGGTTGAATTCGAATTTTC |
| Oph-16-Pgal1/10 | CTATAGTGAGTCGTATTACGGATC |
| Oph-17-tHMG1 | AATTCGAATTCAACCCTCACTATGGTTTTAACCAATAAAACAGTCATT |
| Oph-18-tHMG1 | ATTTGGGCGCGAACGCATGACCGCATGACTCAAGAGAAG |
| Oph-19-ERG13 | TCCGTAATACGACTCACTATAGGATGAAACTCTCAACTAAACTTTGTTG |
| Oph-20-ERG13 | ACATCAATTCATCGGTTGGGCTTAACTT |
| Oph-21- ChrIII | TCATGCGTTCGCGCCCAAATCGGCATC |
| Oph-22- ChrIII | TTAACTATGCGGCATCACTAGTTCATTGGTCACATTACAACCTG |
| Oph-23- ChrIII | GCCCAACCGATGAATTGATGTTACCCTCATAAAG |
| Oph-24- ChrIII | CAGCTATGACCATGATACTAGTATTGCAATTCCTTAGCAACTGG |
| Construct for T_MVD1_-*MVD1-*P_GAL10_-P_GAL1_-*ERG20-T_ERG20_* with ChrIV-1 homologous arm | |
| Oph-15-Pgal1/10 | AGTGAGGGTTGAATTCGAATTTTC |
| Oph-16-Pgal1/10 | CTATAGTGAGTCGTATTACGGATC |
| Oph-25-MVD1 | AATTCGAATTCAACCCTCACTATGACCGTTTACACAGCATC |
| Oph-26-MVD1 | TGAAAGCTAACCTAAAATTTGAG |
| Oph-27-ERG20 | GATCCGTAATACGACTCACTATAGATGGCTTCAGAAAAAGAAATTAGG |
| Oph-28-ERG20 | TATTGTAGTAGTTTTTGTGTTGTG |
| Oph-29- ChrIV | TTAACTATGCGGCATCACTAGTAAACTTTCTTTGAGTCATGGCTC |
| Oph-30- ChrIV | CTCAAATTTTAGGTTAGCTTTCAGACCTAACAATAGTATTCCGGAAC |
| Oph-31- ChrIV | CACAACACAAAAACTACTACAATACTTGATGAATGTGCCTTTCACG |
| Oph-32- ChrIV | CAGCTATGACCATGATACTAGTCTATAACATTTTTATGACTTTGTGCC |
| Construct for T_ERG8_-*ERG8-*P_GAL10_-P_GAL1_-*ERG10-T_ERG10_* with ChrXII-1 homologous arm | |
| Oph-15-Pgal1/10 | AGTGAGGGTTGAATTCGAATTTTC |
| Oph-16-Pgal1/10 | CTATAGTGAGTCGTATTACGGATC |
| Oph-33-ERG8 | GAAAATTCGAATTCAACCCTCACTATGTCAGAGTTGAGAGCCTT |
| Oph-34-ERG8 | TGTTGAAGTTTGTGCACACTTTCAAGCTAAC |
| Oph-35-ERG10 | GATCCGTAATACGACTCACTATAGGATGTCTCAGAACGTTTACATTG |
| Oph-36-ERG10 | CTTCTTCTTCAGCAGCCAAGACATTGATTAAC |
| Oph-37- ChrXII | GAAAGTGTGCACAAACTTCAACACCTTATATCATAACTAATATTTC |
| Oph-38- ChrXII | CAGCTATGACCATGATACTAGTGGTGGTGATGATACAAGACATAC |
| Oph-39- ChrXII | TCTTGGCTGCTGAAGAAGAAGTTAGTTTCTGCC |
| Oph-40- ChrXII | TTAACTATGCGGCATCACTAGTGATGAGATGATATGGACCAAAGAAAG |
| Construct for T_IDI1_-*IDI1-*P_GAL10_-P_GAL1_-*ERG12-T_ERG12_* with ChrXV-1 homologous arm | |
| Oph-15-Pgal1/10 | AGTGAGGGTTGAATTCGAATTTTC |
| Oph-16-Pgal1/10 | CTATAGTGAGTCGTATTACGGATC |
| Oph-41-IDI1 | GAAAATTCGAATTCAACCCTCACTATGACTGCCGACAACAATAGTATG |
| Oph-42-IDI1 | GGAAGGGAACTTTACACTTCTCCAACATTCAAGAGGCCAATAACC |
| Oph-43-ERG12 | GGATCCGTAATACGACTCACTATAGATGTCATTACCGTTCTTAACTTCTG |
| Oph-44-ERG12 | GGATATCCGAAATATTCCACGGCGAAAGCTGAATTGATACTACG |
| Oph-45- ChrXV | GGAGAAGTGTAAAGTTCCCTTCC |
| Oph-46- ChrXV | TTAACTATGCGGCATCACTAGTGGAGTACTATTTGCTAAATATCGTAGAC |
| Oph-47- ChrXV | CCGTGGAATATTTCGGATATCC |
| Oph-48- ChrXV | CAGCTATGACCATGATACTAGTCGTGCAAGTCAGTGTGAC |
| Construct for T_HMG1_-*tHMG1*-P_GAL10_-P_GAL1_-*IDI1*-T_IDI1_ with ChrVI-1 homologous arm | |
| Oph-15-Pgal1/10 | AGTGAGGGTTGAATTCGAATTTTC |
| Oph-16-Pgal1/10 | CTATAGTGAGTCGTATTACGGATC |
| Oph-17-tHMG1 | AATTCGAATTCAACCCTCACTATGGTTTTAACCAATAAAACAGTCATT |
| Oph-49-tHMG1 | TACTTTTCCGACCGCATGACTCAAGAGAAG |
| Oph-50-IDI1 | CGTAATACGACTCACTATAGATGACTGCCGACAACAATAGTATG |
| Oph-51-IDI1 | CTTCCAGAGTGTAATTCCTGAAACATTCAAGAGGCCAATAACC |
| Oph-52-VI | GTCATGCGGTCGGAAAAGTAGCAAATTGCATC |
| Oph-53-VI | TAACTATGCGGCATCACTAGTGGACTCCGCAATAGATCATC |
| Oph-54-VI | TCAGGAATTACACTCTGGAAGTTGGCTTACTG |
| Oph-55-VI | AGCTATGACCATGATACTAGTGAATCTCGGAGTTCGGTG |
| Construct for P_GAL10_-AuoblA-T_ADH1_ with *dpp1Δ* homologous arm | |
| Oph-9-Pgal10 | AACGACCAAAGGCCAGGTTACTGCCAATTTTTC |
| Oph-10-Pgal10 | GAATTCAATTCGAATTTTCAAAAATTCTTAC |
| Oph-11-AuoblA | AATTTTTGAAAATTCGAATTGAATTCATGGAATACAAGTACTCC |
| Oph-12-AuoblA | GCGAAGAATTGTTAATTAAGGAGCTCTTAAACTTTCAACAATTC |
| Oph-13-Tadh1 | TGTAATTCCTGAGCGACCTCATGCTATACC |
| Oph-14-Tadh1 | GAGCTCCTTAATTAACAATTCTTC |
| Oph-56-dpp1 | AAACAGCTATGACCATGATACTAGTACTCGATTTCTGGCGCAGC |
| Oph-57-dpp1 | AACCTGGCCTTTGGTCGTTTGCTATGATTTAATTCT |
| Oph-58-dpp1 | TGAGGTCGCTCAGGAATTACATCCTTTGTCCGA |
| Oph-59-dpp1 | CTTAACTATGCGGCATCACTAGTCTTTGTCAACAGATCGAAGCG |
| Construct for P_GAL10_-AuoblA-T_ADH1_ with *lpp1Δ* homologous arm | |
| Oph-9-Pgal10 | AACGACCAAAGGCCAGGTTACTGCCAATTTTTC |
| Oph-10-Pgal10 | GAATTCAATTCGAATTTTCAAAAATTCTTAC |
| Oph-11-AuoblA | AATTTTTGAAAATTCGAATTGAATTCATGGAATACAAGTACTCC |
| Oph-12-AuoblA | GCGAAGAATTGTTAATTAAGGAGCTCTTAAACTTTCAACAATTC |
| Oph-13-Tadh1 | TGTAATTCCTGAGCGACCTCATGCTATACC |
| Oph-14-Tadh1 | GAGCTCCTTAATTAACAATTCTTC |
| Oph-60-lpp1 | GTAACCTGGCCTTTGGTCGTTCCTTGGTAGAATATGACGAGTTTC |
| Oph-61-lpp1 | CTTAACTATGCGGCATCACTAGTTACTTATACGTCTCCCAATCATGG |
| Oph-62-lpp1 | CATGAGGTCGCTCAGGAATTACAGGCTTTACATATCTTTGGCAAAGG |
| Oph-63-lpp1 | AACAGCTATGACCATGATACTAGTTTCACCGACGGATTCAGAGG |
| Construct for knocking out the *dos2* gene | |
| Oph-64-dos2 | ACTACAACATCGTATTCAATAGGAAC |
| Oph-65-dos2 | ATACGTCATGAAAAGTATGTTTTATCCTAAAACGTCGAAGCAA |
| Oph-66-dos2 | GATAAAACATACTTTTCATGACGTATTATGG |
| Oph-67-dos2 | ATTACCGAGGAATCTGATAGTCC |
| Construct for knocking out the *ynr063w* gene | |
| Oph-68-ynr063w | ACTATTCACCCAGAAGGATATCC |
| Oph-69-ynr063w | CAAAAAATCAGCTAACTGCTTGTTGCGAAC |
| Oph-70-ynr063w | CAACAAGCAGTTAGCTGATTTTTTGACACTTTATTCAGA |
| Oph-71-ynr063w | CCAGACCGATATTCAATTGAGATT |
| Construct for knocking out the *ygr259c* gene | |
| Oph-72-ynr063w | ATGATGATCGGCAAGAAGAC |
| Oph-73-ynr063w | TTGTCATTACCTGGTACTAACCTTCTGAAAATTATGG |
| Oph-74-ynr063w | GAAGGTTAGTACCAGGTAATGACAATAGTTCAATGC |
| Oph-75-ynr063w | CCATTCTCTGCGAAGAAGC |
| Construct for knocking out the *vba5* gene | |
| Oph-76-vba5 | CGTCGAAACTATCTCTTTTCAAAC |
| Oph-77-vba5 | AGAAAATAACTGCGGAATTTCTATCGATTAAGCTCTATACC |
| Oph-78-vba5 | CGATAGAAATTCCGCAGTTATTTTCTTATTTTCATTTTATTTT |
| Oph-79-vba5 | TACTTAGCTGCTGATTTTATCGC |
| Construct for knocking out the *yer134c* gene | |
| Oph-80-yer134c | AATGCAAGGTTGATTAAAATCTTTCA |
| Oph-81-yer134c | GAACTAAGTGCAATAAACGCGTTGAGACTATTTTG |
| Oph-82-yer134c | ACGCGTTTATTGCACTTAGTTCACAGAGC |
| Oph-83-yer134c | CAGGAGATTCTTTTTTGATCTTGAATATC |
| Construct for knocking out the *ypl062w* gene | |
| Oph-84-ypl062w | GAAGAGGAAAAGAAAAAGAAGAAGAT |
| Oph-85-ypl062w | TGCCCACATGGGCCCTTACGTGAGGGGCA |
| Oph-86-ypl062w | GTAAGGGCCCATGTGGGCAAATTCGTAAT |
| Oph-87-ypl062w | CCAACTTACTTAGTAGACGGC |
| Construct for P_GAL2_-*tPOS5*-T_ADH1_ with *dos2Δ* homologous arm | |
| Oph-88-dos2 | AGCTATGACCATGATACTAGTTTTAGTATGGATACCGTACTCC |
| Oph-89-dos2 | TTGGATTAGTACAGCACGCCTTTATCCTAAAACGTCGAAGC |
| Oph-90-dos2 | TGAGGTCGCTCAGGAATTACAACATACTTTTCATGACGTATTATGG |
| Oph-91-dos2 | AACTATGCGGCATCACTAGTATTACCGAGGAATCTGATAGTCC |
| Oph-92-Pgal2 | GGCGTGCTGTACTAATCCAAGGAGGTTTACG |
| Oph-93-Pgal2 | CATTATGAAAGAATTATTTTTTTTATTATGTTAATC |
| Oph-94-tPOS5 | ATAATAAAAAAAATAATTCTTTCATAATGAGTACGTTGGATTCACATTCCCTAAAG |
| Oph-95-tPOS5 | GAATTGTTAATTAAGGAGCTCCTATTAATCATTATCAGTCTGTCTCTTGGTC |
| Oph-13-Tadh1 | TGTAATTCCTGAGCGACCTCATGCTATACC |
| Oph-96-Tadh1 | TAGGAGCTCCTTAATTAACAATTCTTCGC |
| Construct for P_GAL7_-*ADH2*-T_TDH1_-P_PGK1_-*ACS1*-T_ACS1_ with ChrX-1 homologous arm | |
| Oph-97-ChrX | TAACTATGCGGCATCACTAGTTTTGAAGATGAAGCGGTATTTG |
| Oph-98-ChrX | GGCAAATTTCTAAGGCCACTTTTCAATG |
| Oph-99-ChrX | ACATCCTCTACATCGCCTACTTCTTGCCTATTG |
| Oph-100-ChrX | CAGCTATGACCATGATACTAGTATTTGACTGACGAATCGTTAGG |
| Oph-101-Pgal7 | AGTGGCCTTAGAAATTTGCCAGCTTACTATCCTTC |
| Oph-102-Pgal7 | TCTGGAATAGACATTTTTGAGGGAATATTCAACTG |
| Oph-103-ADH2 | ATATTCCCTCAAAAATGTCTATTCCAGAAACTCAAAAAG |
| Oph-104-ADH2 | ACTTAAGGAGTTAAATTTATTTAGAAGTGTCAACAACGTATC |
| Oph-105-Ttdh2 | GACACTTCTAAATAAATTTAACTCCTTAAGTTACTTTAATG |
| Oph-106-Ttdh2 | ATCTGTGCGTGCGAAAAGCCAATTAGTGTG |
| Oph-107-Ppgk1 | GGCTTTTCGCACGCACAGATATTATAACATCTG |
| Oph-108-Ppgk1 | GCGACATTGTTTTATATTTGTTGTAAAAAGTAG |
| Oph-109-ACS1 | TTACAACAAATATAAAACAATGTCGCCCTCTGCCGTAC |
| Oph-110-ACS1 | AGTAGGCGATGTAGAGGATGTGCTGATT |
| Construct for P_GAL2_-*ALD6*-T_ADH1_ with *vba5Δ* homologous arm | |
| Oph-111-Ptef1 | TTCAAAATGTTTCTACTCCTTTTTTAC |
| Oph-112-Ptef1 | CATCTAGAAAACTTAGATTAGATTGC |
| Oph-113-ALD6 | GCAATCTAATCTAAGTTTTCTAGATGATGACTAAGCTACACTTTGACAC |
| Oph-114-ALD6 | GAAGAATTGTTAATTAAGGAGCTCTTACAACTTAATTCTGACAGCTTTTAC |
| Oph-13-Tadh1 | TGTAATTCCTGAGCGACCTCATGCTATACC |
| Oph-14-Tadh1 | GAGCTCCTTAATTAACAATTCTTC |
| Oph-76-vba5 | CGTCGAAACTATCTCTTTTCAAAC |
| Oph-79-vba5 | TACTTAGCTGCTGATTTTATCGC |
| Oph-115-vba5 | GTAAAAAAGGAGTAGAAACATTTTGAAGAATTTCTATCGATTAAGCTCTATACC |
| Oph-116-vba5 | GAGGTCGCTCAGGAATTACATTTCGCCTTTTATACAGACTTC |
| Construct for P_GAL1_-*AuoblB*-T_CYC1_/ P_GAL1_-*AcoblB*-T_CYC1_/ P_GAL1_-*BmoblB*-T_CYC1_ with *ypl062wΔ* homologous arm | |
| Oph-117-Pgal1 | CCCTTTAGTGAGGGTTGAATTCGAATTTTC |
| Oph-118-Pgal1 | GATCCGGGGTTTTTTCTCCTTGACGTTAAAG |
| Oph-119-AuoblB | AGGAGAAAAAACCCCGGATCATGGAAGCCTATTTGCCACA |
| Oph-120-AuoblB | GCTAGCCGCGGTACCAAGCTTTCAGTGGTAAACAACTCTAACAC |
| Oph-121-Tcyc1 | TACAATTTGCCTTCGAGCGTCCCAAAAC |
| Oph-122-Tcyc1 | GCTTGGTACCGCGGCTAGCTAAGATCCGCTCTAACCGAAAAG |
| Oph-87-ypl062w | CCAACTTACTTAGTAGACGGC |
| Oph-84-ypl062w | GAAGAGGAAAAGAAAAAGAAGAAGAT |
| Oph-123-ypl062w | ACGCTCGAAGGCAAATTGTAGACCATGTGGGCAAATTCG |
| Oph-124-ypl062w | GAATTCAACCCTCACTAAAGGGGCCCTTACGTGAGGGGCAG |
| Oph-125-AcoblB | AGGAGAAAAAACCCCGGATCATGGAAGCTAGTTTGCAT |
| Oph-126-AcoblB | TAGCTAGCCGCGGTACCAAGCTTTATCTAAAAATAACTCTAAAACCTTTAGATTC |
| Oph-127-BmoblB | AGGAGAAAAAACCCCGGATCATGTCATCCATTCAATTACAAAAGTAC |
| Oph-128-BmoblB | TAGCTAGCCGCGGTACCAAGCTTTAGTTAAACTTAACTTTAAAACCCTTAG |
| Construct for P_TDH3_-*AuoblC*-T_CYC1_ with ChrVII-1 homologous arm | |
| Oph-129-Ptdh3 | ATCATTATCAATACTCGCCATTTC |
| Oph-130-Ptdh3 | TCGAAACTAAGTTCTGGTGTTTTA |
| Oph-131-AuoblC | ACACCAGAACTTAGTTTCGACGGATTCTAGAATGCCATTGCCAAAATCCTTC |
| Oph-132-AuoblC | CTCGAGGTCGACGGTATCGATAAGCTTTTACAAACCTTCAACCAAAGC |
| Oph-133-Tcyc1 | ATACCGTCGACCTCGAGTCATGTAATTAGTTATGTCACGC |
| Oph-121-Tcyc1 | TACAATTTGCCTTCGAGCGTCCCAAAAC |
| Oph-134-ChrVII | CAGCTATGACCATGATACTAGTGAATTCATCGACATAATGCACCTCAG |
| Oph-135-ChrVII | TGGCGAGTATTGATAATGATTGGATTAGTACAGCACGCCACACTGACTATG |
| Oph-136-ChrVII | GACGCTCGAAGGCAAATTGTATAGAACACTGTAGAGTAGCGC |
| Oph-137-ChrVII | TTAACTATGCGGCATCACTAGTGAATTCGCTCTTCTTACTAATAAGTTGCC |
| Construct for P_GAL1_-*BmoblB*-T_CYC1_ with ChrXI-1 homologous arm | |
| Oph-127-BmoblB | AGGAGAAAAAACCCCGGATCATGTCATCCATTCAATTACAAAAGTAC |
| Oph-128-BmoblB | TAGCTAGCCGCGGTACCAAGCTTTAGTTAAACTTAACTTTAAAACCCTTAG |
| Oph-118-Pgal1 | GATCCGGGGTTTTTTCTCCTTGACGTTAAAG |
| Oph-138-Pgal1 | CAAAAATTCCACTGGAACTTTCAGTAATACGCTTAAC |
| Oph-121-Tcyc1 | TACAATTTGCCTTCGAGCGTCCCAAAAC |
| Oph-122-Tcyc1 | GCTTGGTACCGCGGCTAGCTAAGATCCGCTCTAACCGAAAAG |
| Oph-139-ChrXI | AACTATGCGGCATCACTAGTCATATGAGAAATCCCGTAGCTGAAG |
| Oph-140-ChrXI | TTACTGAAAGTTCCAGTGGAATTTTTGAGACTTTATTGG |
| Oph-141-ChrXI | ACGCTCGAAGGCAAATTGTAGATATTTCGAATGC |
| Oph-142-ChrXI | GCTATGACCATGATACTAGTGAATTCGTCTTTACACGTTGGCCAC |
| Construct for P_TEF1_- *Au_9779_CPR*-T_PRM9_/ P_TEF1_- *Au_674_CPR*-T_PRM9_/ P_TEF1_- *NCP1*-T_PRM9_ with ChrIV-2 homologous arm | |
| Oph-143-ChrIV | TAACTATGCGGCATCACTAGTGAGCTCACGAAAGAAGCTGAATCTTTAC |
| Oph-144-ChrIV | TATACCTTATTGTTGTTAATATGTCCTTAATATGTCCAGTCGGAGTG |
| Oph-145-ChrIV | GGTATATATTGGCTATTCTTATGGTTAATGTCACGAGATG |
| Oph-146-ChrIV | AGCTATGACCATGATACTAGTGAGCTCTTATCAGTCTCTGCATTTTCAG |
| Oph-147-Ptef1 | GGACATATTAACAACAATAAGGTATATTCAAAATGTTTCTACTCCTTTTTTAC |
| Oph-112-Ptef1 | CATCTAGAAAACTTAGATTAGATTGC |
| Oph-148-Au_9779_CPR | GCAATCTAATCTAAGTTTTCTAGATGTCTCTATCTACTTTAACGCC |
| Oph-149-Au_9779_CPR | GTGTCTCCCGTCTTCTGTCTAACCCCAAACATCTTCTTGCC |
| Oph-150-Au_674_CPR | GCAATCTAATCTAAGTTTTCTAGATGGCTCAATTGGATACTTTGG |
| Oph-151-Au_674_CPR | GTGTCTCCCGTCTTCTGTCTAAGACCAGACATCTTCTTGATAAG |
| Oph-191-NCP1 | GCAATCTAATCTAAGTTTTCTAGATGCCGTTTGGAATAGACAAC |
| Oph-192-NCP1 | GTGTCTCCCGTCTTCTGTCTACCAGACATCTTCTTGGTATC |
| Oph-152-Tprm9 | TAGACAGAAGACGGGAGACAC |
| Oph-153-Tprm9 | AACCATAAGAATAGCCAATATATACCTTGTATTGCCTATG |
| Construct for P_TEF1_- *Au_9779_CPR*-T_PRM9_-P_PGK1_-*AuCYB5*-T_IDP1_ with ChrIV-2 homologous arm | |
| Oph-143-ChrIV | TAACTATGCGGCATCACTAGTGAGCTCACGAAAGAAGCTGAATCTTTAC |
| Oph-144-ChrIV | TATACCTTATTGTTGTTAATATGTCCTTAATATGTCCAGTCGGAGTG |
| Oph-145-ChrIV | GGTATATATTGGCTATTCTTATGGTTAATGTCACGAGATG |
| Oph-146-ChrIV | AGCTATGACCATGATACTAGTGAGCTCTTATCAGTCTCTGCATTTTCAG |
| Oph-112-Ptef1 | CATCTAGAAAACTTAGATTAGATTGC |
| Oph-154-Ptef1 | ATCTGTGCGTGATCTTCAAAATGTTTCTACTCCTTTTTTAC |
| Oph-148-Au_9779_CPR | GCAATCTAATCTAAGTTTTCTAGATGTCTCTATCTACTTTAACGCC |
| Oph-149-Au_9779_CPR | GTGTCTCCCGTCTTCTGTCTAACCCCAAACATCTTCTTGCC |
| Oph-152-Tprm9 | TAGACAGAAGACGGGAGACAC |
| Oph-153-Tprm9 | AACCATAAGAATAGCCAATATATACCTTGTATTGCCTATG |
| Oph-155-Ppgk1 | TTTGAAGATCACGCACAGATATTATAACATCTG |
| Oph-156-Ppgk1 | AGCAGACATTGTTTTATATTTGTTGTAAAAAGTAG |
| Oph-157-AuCYB5 | CAACAAATATAAAACAATGTCTGCTGGCAAAGAG |
| Oph-158-AuCYB5 | CGTAAATTCGATTATGCTTGTTCAGCTGCTGC |
| Oph-159-Tidp1 | CTGAACAAGCATAATCGAATTTACGTAGCCCAATC |
| Oph-160-Tidp1 | GGACATATTAACAACAATAAGGTATATATATTTATGATAACAG |
| Construct for P_TDH3_-*PDR1*-T_CYC1_/ P_TDH3_-*PDR3*-T_CYC1_/ P_TDH3_-*PDR5*-T_CYC1_/ P_TDH3_-*PDR10*-T_CYC1_/ P_TDH3_-*SNQ2*-T_CYC1_ with ChrX-2 homologous arm | |
| Oph-161-ChrIV | CATTTTGAAGATCTACGAGCGGAGGAATAGTATG |
| Oph-162-ChrIV | ACAGCTATGACCATGATACTAGTCCGTCTATGAGGAGACTGTTAG |
| Oph-163-ChrIV | TTAATTTGCTTTCGCCTTTTATTGACGAAGGCTAAGGTCAC |
| Oph-164-ChrIV | TAACTATGCGGCATCACTAGTTGGATGGTAGGGAGAGTACG |
| Oph-165-Tcyc1 | ATAAAAGGCGAAAGCAAATTAAAGCCTTCGAGC |
| Oph-166-Tcyc1 | AGCTTATCGATACCGTCGACCTCGAGTCATGTAATTAGTTATGTCACG |
| Oph-167-Ptdh3 | CCGCTCGTAGATCTTCAAAATGTCATTATCAATACTCGCCATTTC |
| Oph-168-Ptdh3 | GATCCACTAGTTCTAGAATCCGTCGAAACTAAGTTCTGGTG |
| Oph-169-PDR1 | GATTCTAGAACTAGTGGATCCATGCGAGGCTTGACACCTAAG |
| Oph-170-PDR1 | GTCGACGGTATCGATAAGCTTTTAACTATCTGGATAAACGTCGC |
| Oph-171-PDR3 | GATTCTAGAACTAGTGGATCCATGAAAGTGAAGAAATCAACTAG |
| Oph-172-PDR3 | GTCGACGGTATCGATAAGCTTTCATAAGAAGGGATATGAAGTATTG |
| Oph-173-PDR5 | GATTCTAGAACTAGTGGATCCATGCCCGAGGCCAAGCTTAAC |
| Oph-174-PDR5 | GTCGACGGTATCGATAAGCTTTTATTTCTTGGAGAGTTTACCGTTC |
| Oph-175-PDR10 | GATTCTAGAACTAGTGGATCCATGTTGCAAGCGCCCTCAAG |
| Oph-176-PDR10 | GTCGACGGTATCGATAAGCTTTTATTTCTTTAATTTTTTGCTTTTCTTTG |
| Oph-177-PDR10 | GATTCTAGAACTAGTGGATCCATGAGCAATATCAAAAGCACG |
| Oph-178-PDR10 | GTCGACGGTATCGATAAGCTTTTACTGCTTCTTTTTCCTTATG |
| Construct for P_GAL2_-*CAT2pex-T_PDC1_* with ChrII-1 homologous arm | |
| Oph-179-ChrII | AACTATGCGGCATCACTAGTGAATTCGACTTTTGAACTTCGATGACG |
| Oph-180-ChrII | TAAGGATTCGCGGTGAAGGTGTAGATAAACTAGGTTAC |
| Oph-181-ChrII | ACTAGACACCTCGATACAAATATGTTCATATCACGGCATACG |
| Oph-182-ChrII | GCTATGACCATGATACTAGTGAGCTCGGTAAGTTCTTAGGACTTCTTG |
| Oph-183-Pgal2 | ACCTTCACCGCGAATCCTTATGTACTAATCCAAGGAGGTTTACG |
| Oph-184-Pgal2 | GAATGACAGATCCTCATTATGAAAGAATTATTTTTTTTATTATGTTAATC |
| Oph-185-CAT2 | TAATTCTTTCATAATGAGGATCTGTCATTCGAG |
| Oph-186-CAT2 | CCAGGTAAACTAACTTTGCTTTTCGTTTATTCTC |
| Oph-187-PEX15 | AAAGCAAAGTTAGTTTACCTGGCTGGTTCATTTTGGGC |
| Oph-188-PEX15 | TCGCTCATATACTCGCTAGAAGTTTTAG |
| Oph-189-Tpdc1 | TCTAGCGAGTATATGAGCGATTTAATCTCTAATTATTAGTTAAAG |
| Oph-190-Tpdc1 | ACATATTTGTATCGAGGTGTCTAGTCTTCTATTAC |

**Table S2.** The gRNA sequences used for CRISPR-Cas9 system.

| gRNA | Sequence (5’–3’) |
| --- | --- |
| *gal80*Δ | ATAAGGCTGCTGCTGAACGT**GGG** |
| *dpp1*Δ | ACACGTTCAGTTGTCGCATA**AGG** |
| *lpp1*Δ | TCAACTTTAAGGCGCCTGTT**AGG** |
| *dos2*Δ | ATAGAACAGAAGCCGAACTA**AGG** |
| *ynr063w*Δ | AACGAGCTTCACTCCGTACC**AGG** |
| *ygr259c*Δ | AGTAGCCATAATTTCCCGTT**CGG** |
| *vba5*Δ | CAATAACAAAGCAAAGCGTT**TGG** |
| *yer134c*Δ | TAAGATCACCTAGTATACGT**GGG** |
| *ypl062w*Δ | TTCGACATAAATGAGGGGCA**TGG** |
| ChrI-1 integration | GTATCACAACCGACGATCCG**AGG** |
| ChrIII-1 integration | TGGCCCTGATAATAGTATGA**GGG** |
| ChrIV-1 integration | TGTCAAAAAATGGAGTAGTA**TGG** |
| ChrXII-1 integration | AAGATAGGTAAATAAACGCG**GGG** |
| ChrXV-1 integration | CCAATGCTAGTAGAGAAGGG**GGG** |
| ChrVI-1 integration | TTAAGGAAATACCCATCCGT**TGG** |
| ChrII-1 integration | ATTAACTTAGCTATGTAGGC**CGG** |
| ChrX-1 integration | GACACATTAGTCTCGTATGT**CGG** |
| ChrVII-1 integration | CTAATCAAATGTATCGTGAC**TGG** |
| ChrXI-1 integration | TAAAAGCATTCACTGCCCGC**AGG** |
| ChrIV-2 integration | TCATCATTGCTATATCGGCA**AGG** |
| ChrX-2 integration | CTTTCAACAACCGGGCGACT**AGG** |

**Table S3**. Synthesized genes list (All genes were codon optimized for *S. cerevisiae*)

| Gene | Sequence (5'-3') |
| --- | --- |
| *AuoblA* | ATGGAATACAAGTACTCCACCATTGTTGATTCCTCTAAGTGGGATCCAGAAGGTTTGATTGAAGGTATTCCATTGAGAAAGCATGAAGCAGGTGATTTGGAAGAAGTTGGTAGTTTTAGAGTTCAAGAAGATTGGAGAAGATTGGTTGGTCCAGTTGAAAATCCATTCAGAGGTAGTTTGGGTCCAGAAATTAGTTTCATTACCTACACCGTTCCAGAATGTTTGCCAGAAAGATTGGAAGCAATTTCCTACGGTTTGGAATACGGTTTCTTGCATGATGATGAAATTGATACCAAGATTGAAGAAGCTGAATTGGATGATGTTGGTGCTGCTTTGGCTCAAGGTGGTTCAACTGGTAAAATTCAAGAAGGTACCAAGTCCTCAGGTAAAAAGAAAATGGCTGCTCAATTGTTGAGAGAAATGATGGCTTTGGATCCAGAAAGAGCTATGACTTTGGCTAAGAGTTGGGCTCAAGGTGTTCAACATAGTGCAAGAAGAGTTGAAGAAAAGGATTGGAAGTCCTTGGATGAATACATTCCATTCAGATGTATGGATTTGGGTTACATGCATTGGCATGGTTTGGTTACTTTCGGTTGTGCTATTACAGTTCCAGAAGAAGAAGAAGAAGAAAGAAGAACCTTGTTGGAACCAGCTGTTATTGCTTGTTTGATGACTAACGATTTGTTCTCCTACGAAAAGGAAAAGAACGATAACAACCCACAAAACGCAGTTGCAGTTATTATGAAGATTCATAAGTGTTCCGAAGAAGAAGCTAGAGATATTTGTAAGCAAAGAATTAGATTGGAATGTAGAAAGTACGCTAGAATTGTTAAGGAAACCTTGGCTAGAACCGATATTTCCTTGGATTTGAAGAGATACATTGAAATTATGCAATACACCGTTTCCGGTAACTGGGCATGGAGTACTCAATGTCCAAGATATCATGCTGATGCTAAGTTCAACGAATTGCAAATGTTGAGAGCTGAACATGGTGTTGCTAAATACCCAGCAAGATACAGTTTGGAAAACAGAAAGAACGGTGCAAACGGTGTTAATGGTGTTAATGGTATTAACGGTGTTAACGGTGTTAACGGTGTTAATGGTAAGAGAAAGAGATCCGGTGAAGAAACAGCTGATGATGCTAGAACAAACGGTAATGGTATTAAGAAGCCAGCTCATGTTTTGGAATACAGAGATTCATTGGTTTTGGAAGATATTGTTGCTTTGTCCTTGGATTGGAACTTGCCAGATTTGTCTGATGGTGTTGTTGTTCAACCATACAAGTACTTGACCTCTTTGCCTTCTAAGGGTTTTAGAGATCAAGCTATTGATTCCTTGAACACCTGGTTGAGAGTTCCAACTAAAACTACTAAGATGATTAAGGATGTTATTAAGATGTTGCATTCCGCTTCCTTGATGTTGGATGATATTGAAGATAACTCCCCATTGAGAAGAGGTAAACCTTCTACTCATGTTATTTACGGTAACGCACAAACCATTAACTCCGCTACATATCAATACACCGAAGCTACTGGTTTGGCTGCTAGATTGCCAAACCCAACTTCTTTGAGAATTTACTTGGAAGAAGTTCAACAATTGTACATTGGTCAATCCTACGATTTGTACTGGACCCATAATGCATTGTGTCCATCTATTCCAGAATACTTGAAGATGGTTGATCAAAAGACCGGTGGTTTGTTTAGAATGTTGACTAGATTGATGGTTTCCGAATCCCCAGCTAGATCTTCTATTTTGGATCAAACCTTGTACCCATTGTCCCATTTGATTGGTAGATTCTTCCAAATTAGAGATGATTACCAAAACTTGGCTTCCGCTGAATATGCTAGACAAAAAGGTTACGCTGAAGATTTGGATGAAGGTAAGTACTCTTTCACCTTGATTCATTGTATTAACACCTTGGAAGCTGAAGCATCTTTGGCTTCTGAAAAAATGGCTTTGAGAGCTTTTTTGATTAAAAGAAGAGTTGATTCTTCTTTGTCTAATGAATCTAAAAGAGAAGTTTTGGATATTATGAAAAAAACTAAATCTTTGGAATATACTTTGGGTGTTTTGAGAGCTTTGCAAGCTGAATTGGAAAAAGAAGTTGATTCTTTGGAAGCTAAATTTGGTGAAGAAAATTTTTCTTTGAGAATGATGTTGGAATTGTTGAAAGTTTAA |
|  |  |
| *AuoblB* | ATGGAAGCCTATTTGCCACAAGCTAGTAGAGAAGCCTTGGCTAGATTATTGCCATCTCAAGCCTCTACTGCTAGATTGGCTGAAGTTCAAGCCTACTTGACTAGAGATGTTGCTGTTAGAGCTGGTTTGGCTGTTGCTGCTATCTATATGGTTTATTTGTCCGGTTTGGTCATCTACAGGTTGTACTTTTCTCCATTGGCTAAATTTCCAGGTCCAAGAATTGCTGCTGTTACTGGTTATTATGAGTTGTACTACGATGTCATCCATAAGGGTCAGTACATCTTCCAAATCGAAAAGATGCATGATAAGTACGGTCCAATCGTTAGAGTTAACCCATTCGAATTGTCCATCAGAGATTCCGATTACTATGACGAGTTGTACGTTATGGGTAACATCAGAAAGACCGATAGATACGAAGGTTTCGTTTCTGGTGTTGTTGATTTCGAAGGTTCTCATTTGGCTACTATTGCTCATGACTTGCACAGAAAGAGAAGAAAACCATTGGATGTCTACTTCTCCAGACAAGGTGTTACTAGAGTTGAACCTATGGTTGCTGATTTGACTGCAAGATTGGTTGTTGACAGATTGGAGTCTTTTAAAGGTACTGGTAAGGTCGTTAGATTGGATCATGCTTTTACTGCTTTTTCCGGTGATGTCATTAACAGAATCTGTGTTGATAAGCCCTCCGAAACCTACATTGATGATGATGAATTTTCCCCATGGTGGTTCGATTTGTTTCATATTGGTGCTGTTTCTCTGCCTTTGTTTATGGGTTTGCCATGGTTGATTCATCTGATTAGATTACTGCCAGTCGGTATCGTTTCTACCATTAACTCTTCAGCTGGTTCCTTCAACAAGTTCAAGATCATGTGTGATGAACATTTGACCGAAGCCAAAAGGGAAAAGTCTTACACTTCTAAAACTCAACCAGCCTCTGGTAGATTGACTTTGTTTAGACATTTGGTCGATTCTGATTTGCCAGCTTCTGAATTGACTGACTCTAGATTGTCTAGAGAAGCTCAAGTTTTGATCGGTTCTGGTACTATGACTACTGCTGGTACAATGGGTTTTTTGTGCTACTACATTATGTCTAACCCAGCCATCAGACAAAGATTGGCAGATGAATTGGGTCCAGTTATGAAGGATTATCCAAGAACTAAGCCAACTTGGGCTGAATTGGAAAAGTTGCCATATTTCCAAGCCGTCATCAAAGAAGGTTTGAGATTGTCTTACGGTACTATGCATCGTAGACCAAGAGTTTCTCCAACTCAACCTTTGTTGTTTAAGGATTGGGTTATTCCAGCTGGTGTTCCAGTTGGTATGTCTGCTTATTTTCAACATAGAGATCCAAACGTCTACCCAAGACCAATGGAATTTTTGCCAGAAAGATGGTTGGGTGAAGTTACTCCAGCTATGTACAAGAACTACATCCCATTTTCTAAGGGTTCCAGACATTGCTTGGGTATGAATTTGGCTTACTGCGAACTGAACTTGATTATTGCTGCTTTGTTCAGACCAGGTGCTCCAAAATTTGACTTGTATGGTACTGATGAATCCGATGTTAGACCAGCTCATGATTTGATAGTTCCAATGCCAAGATTGGATTCCTTGGGTGTTAGAGTTGTTTACCACTGA |
|  |  |
| AcoblB | ATGGAAGCTAGTTTGCATTTTCCAATTGATGCTAGTTTGAGAGCTGCTGTTGCTGTTATTTCTGCTGGTGCTTTTTATTTGTTGAATTTGGTTATTTACAGATTGTTCTTGTCCCCATTGGCTAAATTTCCTGGTCCAAAATTGGCTGCTGTTACTTCATGGTATGAATTGTATTACGATTTGGTTCATAAGGGTAAGTATTTGTTTGAAATTGAAAAGATGCATGATAAGTACGGTCCAATTGTTAGAATTAATCCATTTGAATTGTCCATTAGAGATTCTGAATACTATGATGAATTGTACGTTGCTGGTAGTGTTAGACCTACTGATAGATATGAAGCATTTGTTGAAGGTATTGTTGATTTTAAGGGTTCCCATATTGCTACTATTGAACATGATTTGCATAGAAAAAGAAGAAAGCCATTGGATCCATATTTTTCTAGATTGGGTGTTTCTAGATTGGAACCAATGTTGGGTGAATTGACAGAAAAGTTGATTGTTAACAGATTCGAATCTTTCAAGAGAACTGGTAAGGTTGTTAGATTGGATCATGCTTTTACAGCTTATTCTGGTGATGTTATTAACAGATTGTGTATGGATGATCCACCAGATGTTTTGGTTGATGATCCAGAATTTTCACCATGGTGGTATAATATGTTTCATAATGGTATTGCTACCTTGCCTTTGTTTATGGGTTTGCCATGGTTGATTCATGTTGTTAGATTAATTCCTGTTTCCATTTTGGCTAAATTGGATCCTGGTACTCAAACTTTTAATAAATTCAAGATGATGTGTGATGATCATTTGAGAGTTGCTAAAAGAGAAAAAGCTGCTCAAGGTTCCAAGGATACTTCTATGATGGATGCTAGACCTACTATTTTCAGACATTTGTTGAACAGTGATTTGCCACCTTCTGAATTAACTGATGATTTGTTGAGTAAGGAAGCTCAAGTTTTGATTGGTACTGGTACTATTACTACTGCTGGTTCTTTGTGTTTTATTTGTTATCATATTGTTGTTAACCCAGCAATTAAGAAGAGATTGCAAGAAGATTTGAAGTTGATTATGGCTAATTACCCTGCAAAAAAACCAACTTGGGCTGAATTGGAAACTGCTACTTATTTGCAAGCTGTTATTAAAGAAGGTTTGAGATTGTCTTTTGGTACTATGCATAGAAGAACTAGAGTTTCTCCAAAACAACCATTGCAATTTAGACAATGGACTATTCCAGCTGGTGTTCCAGTTGGTATGTCTGCTTATTATGCTCATAGAGATCCATCTGTTTTTCCAAGACCAGATGAATTTTTGCCAGAAAGATGGTTGTCTAATGTTACTCCAGAAATGTCTAGAAATTATGTTCCATTTTCTAGAGGTTCTAGAAGATGTTTGGGTATGAATTTGGCTTATGCTGAAATTAATCATGTTATTGCTACTTTGTTTAGACCAGGTGGTCCAGATTTTAAATTGTATGAAACTTCTGAAAAAGATGTTAAACCAGCTCATGATTTGATTGTTCCATTGCCATCTTTGGAATCTAAAGGTTTTAGAGTTATTTTTAGATAA |
|  |  |
| *BmoblB* | ATGTCATCCATTCAATTACAAAAGTACTTAGATTTGTTGCCAGAAAACGCTATCTTGAATGCTGGTATTGCCATTGCTGGTTTATCTGCTGCTTATGCTATCGGTTTGGTCATCTACAGATTGTACTTGTCTCCTATTAGTAAATTTCCAGGTCCAAAGATTGCAGCTGCTACTTTCTGGTACGAATTATATTACGACGTTATTCATAAGGGTCAATACTTCCATAAGATTGAAGAAATGCATGAAAAATATGGTCCAATCGTTAGAATTAACCCACACGAATTATCTATTAGAGACCCAGATTACTACGATGAATTGTACGTGTCAGGTTCTGTTAGACCTTCTGATAGATATGAAGGTTTCGTTAATGGTGTTGTTGACTTCGAAGGTTCACATTTGGCTACCGTAGCTCATGAATTGCATAGAAAGAGAAGAAAGCCATTGGACCCATACTTTTCTAGAGCTGGTGTTAACAGACTAGAACCAATGGTAGCAGATTTGACCGAAGAATTAGTTGTTAAGAGATTTGAAGAGTTTAAGGGTACTGGTAAAGTTGTTAGATTGGATCACGCTTTCACTGCTTACTCAGGTGACATTATTTCCGCTTTATGTATCGATGAACCACCTCATTTCACTTCAACTCCTGATTTCACTCCATCCTGGTTCGATTTGTTTCATTCTGGTGTTGTTACCTTACCTCTTTTTATGGGTTTGCCATGGTTGATTCACTTAATTAGATTAATTCCAGAATCTATTTTGGCTGTTATTGATCCAGGTGCTCAAAACTGGAATACCTTTAGAATGATGTGTTATGACTCAATTAAGGATACTAAAAGAGAAAAGGGTGCTCAACCAACTAAGGATACTTCTTTGTTAGGTAGGCCAACTTTGTTCCGTCATTTGGTTAACTCTGACTTGCCTGCTTCTGAATTGTCCGATGAACGTTTATTAAGAGAAGCCCAAGTCTTGATTGGTTCAGGTACTATGACTACTGCTGGTACTATGTGTTTCTTAGTCTATTACATTAAGTCTAATCCTGAAATTCATAGAAGATTGACTGAAGAATTGAAGCCAATCATGGAAGGTTACCCACATAAGAAGCCATCCTGGGCTGAAATTGAAAAAGCTGAGTATCTGCAAGCTGTTTTGAAGGAAGGTTTGAGATTGTCTTTCGGTACCATTCATCGTAGACCTAGAGTTTCCCCAAATCAACCATTGCAATTCAAGGAATGGGTCATCCCAGCTGGTGTCCCAGTTGGTATGTCTGCATACTTCCAACATACCGATCCAAAGATTTTCCCTAACCCACATGAATTCAACCCAGATAGATGGTTGTCTAACGTCACTCCAGCTATGAAGAAGAATTACGTTCCATTTTCTAAGGGTTCTAGACATTGTTTGGGTATGAACTTGGCTTACTGTGAATTGAACTACATTATCGCTACTATGTTCAGACCAGGTGCTGTTGATTTTGATCTATTTGAAACTACTGAATTAGATGTTAAACCAACCCACGATATGGTTGTTCCATTACCATCTTTAAAATCTAAGGGTTTTAAAGTTAAGTTTAACTAA |
|  |  |
| *OblC* | ATGCCATTGCCAAAATCCTTCTTCGCTGGTGTTTTGGCTTACTTGGCTTCTATTGCTTTGTCTTCTCCTACTCCTCAATCTCCTGATTTGTCTGCTTTTCAACCTGGTGATATTATTAGAAGAGATGTTGCTGTTGTTGGTGGTGGTTCTTCTGGTACTTATTCTGCTATTTCTTTGGTTGATAAGGGTAGATCCGTTATTGTTATTGAAAAGCAAGGTAGATTGGGTGGTCATACTGCAACTTATGTTGATCCTGCTACTGGTACTACTACTGAAGTTGGTGTTCAAATTTATCATAACTACACTATTGTTAAGGATTACTTCGCTAGATTTGATATTCCTATTATTAACTCCCCAGGTTTTTATCCATCTGGTAATAATTTTGATTACAGAACTGGTGAAAGAGTTACTTTAACTCCACCATCTGCTTCTGAAGTTTCTTTGGCATTTGCTGCTTATTCTGAACAATTGGCTAAGTATCCAGGTTTGAATAATGGTATTTACTTGCCTGATCCAGTTCCAGAAGATTTGTACTTACCTTTTGGTGAATTTGTTGAAAAGTACGGTTTGCAAGCTGCTGTTCCATCTATGTATCATTACAATCCAGGTGTTGGTAACATGTTGGATAATCCAACTTTAGAAGCATTTAGATACTGGTCTTTGCATGGTATGGTTCAATCTATTGCTACAGGTTTTTTGATTACTGAAAGAAGAAACTCTTCCGAATTGTATGCTAAAGCTGTTGAAGAATTGAGTGAAACTGGTTCAGTTTTGTTGAATTCTCAAGTTGTTACCGCTGTTAGAAAAGAAGGTACCGCTGGTGTTACATTGATTGTTGATACTCCAACTGGTAAGAAGTTGATTTTGGCTAAGAAGTTGTTGGTTGCTGTTCCTCCAAAATTAGATATTGTTAGACCTTTGGATTTGTCTGATGATGAAAGTACTTTGTTTTCTAAGTTCATTTCCGCTGGTTATTATGCTGGTGCTTTGGTTGATACTTCTTTTTCTGAAGAAACTTCCCATTCTAATGCTATTCAAGATGCTCCTTATAATTTGCCAGCTTTGCCAGCTGCTTATAACTTTTCTCCATCTAGGGTTAGAGGTGTTCAAATTTTCCATTATGGTACTCCTCAATCCGAAGAAACCTTTCCATTAACTGATGATGAAGTTGAATCTCATGTTATTGATACTGTTAGAAGAATTCAAGAACAAAACCCAAATTTGTACAACATTACCGATCCAAAAGTTGTTGCTTTTTATCCACATGCTCCATATAGTTTGCAAGTTTCTGGTGAAGATATTAGAGATGGTTTTTATGAACAATTGTACGCTTTACAAGGTCAAAGAAACACTTATTGGACCGGTGCTGCTTGGAGAGCTGAAGATTCTTCTTTGTTGTGGAGATTTTCTGAAGAAGTTGTTTTGCCTGCTTTGGTTGAAGGTTTGTAA |
|  |  |
| *Au_9779_CPR* | ATGTCTCTATCTACTTTAACGCCTCCAATCTCAATCTCTCCATCTACTTTGTCTTTCTTAACTCAACACTTACAATTGCATTTGCAAACTCAACCATCTAGTCCAGCTGATTATATTGCATTGACATTTCTGTCTCTATTAGGTGCTACTTACTTGTCTAGGGGTATATTGTGGGATACTCCAGATCCATACAGACATCTGTTGTACGAAAGACCACAATTAAAGTACGGTAATGGTGCAAATGGTACAAACAACGGTTCTGGTGTTGCTGGTGATAATGCTCATCAAGCTACAAGAAACGTTGCTAGAAAATTGGAAGAAACCGGAGCATCTATCGTTATCTTCTGGGGTAGTCAATCTGGTACGGCAGAATCTTTGGCTCATAGATTAGGTAGAGAAATTACAGCCAGATTTAAGGGTGAAGGTCAAGTATTGGGACATGGTCAAGGTGCATTAATCGCTGATTTGTCAGACTATGATCCAGAAAGTGTGGCTGAAGTTTCCAGATTACAATCTAAACTGGTAATTTTCGTGTTGTCCACATACGGTGAAGGTGACCCAGCTGACAATACAATCGAGTTCTGGGATTGGTTGAATGCAGTCGAGAAGAAGGCTGCTAAAGAGAATCAATCCGAAGGTAAGAAATTGTTTGACGGTTTGAGATACTTCGCCTTTGGTTTGGGTAATTCCAACTACAAGTTCTATAACAGAGTTATCGACAGAGTTGCTGAAGTTCTGGATAATCATGGTGCTAATGCATTGTTGCCTATCTCAAGAGCTAATGATGCTAATGGCACAACACAAGAAGATTTCATCTCTTGGAAGGAGGATTTATTCACCTACTTTAGAGAAGAATTAGGCTTTGCTCAAAGTGAACCAGTTTATCAACCAGCAATCTTTATCACACAAGATGCTAACATTGAGGCTTCTTCATTGCATAGAGGTGAACCAAGACATTCACCTTCTCCAACTGCTATTCCAATTACTGGTATCAGACAACTGTTTGAACCATCTTCAGACAGACATTGCTTGCATATCGATCTTGACATCTCAGGTACTCCAGATGTGGTTTACAAGACAGGCGATCATTTGGGTGTTTGGCCATCAAATCCAGATGAAGAAGTGGACTTGTTGTTACAAATGTTGGGTAGAGCACATGAAGGTGACGTTCCAATTTCTATTTCACCAGTTGAAGGTGCTGATGTCGAAGATATGTCCAAGAAGGTGCCTTCTCCAACAACTTTACTAGTTCTGTTAAGACATTACCTGGAAATCACTGCTTTGATACCAAGAGACGTGTTGGCTACTTTAGCTCCATTTGCTCCAACACCAGAAGCTAAGACCTTGCTTACTACTTTGGCTAACTCCAAAGACTCTTATGCTGAATTTACTAGAAGAAACCATCTTACCTTAGCTAGAATCTTGGCTGCTGCATCTGGTGGTCAACCTTGGAGAAACCTTCCACTTAGTTACATTGTCGAAACTTTGCCACCATTGCAACCAAGATACTATTCCATTTCTAGTTCATCAGTTGTTAGTCCAAGAAAGATTTCACTAACTGTCTTAGTTCATAAGACTGCTTTACCAGAGAATGCTACAGTTTCAATTCCAGGTTTGACTTCAACATACTTGTTATCACTTTCAGATTTACCTTCCCCTTCTTCCAGTTCTGAAGGTAAGGCTATCTTGCAATCAACTTATACTAACACCTTGTTACTGCCTATTGCTCCATCACCTTCTACACCTCAACCTCAGAAGAGAATCTTCTCTCATTTGAGAAGATCAACATTTAAGTTGCCAAGAACTGCTACCACTCCATTGATCTTGATCGCAGCAGGCACTGGTTTAGCACCTTTAAGAGCTTTCTTGCATGAAAGACGTCAATTACTGAAGATTGGTAGAGATGTTGGTGATATGTTGTTGTTCTTCGGTTGTCGTAGACCAGATGAAGATTTCATTTACAGAGATGAACTTGCTGAAATGGAGAATGCATTTGGTAAACAGTTAAAGATTGTTACCGCTTTCTCTCGTTTAGATACTGATGGTGGTGGTAAGAGAGGTTACGTTCAAGAAAGAGTCGTTGAGTTTAAAGAAGAAGTTAGAGAACTATTAGTTGAAAGAACTGGTAACTTGTACATCTGTGGTAGAGCTGGCATGGCTAGAGAAGTTGAGAAGAGAGTTACAGGTTTTCTAGCAGAAGGTGAGAATGGTAATGGTATGGGTTCAGTCAAAGCTGCCGAAGAGTGGGTTAGAGGTGTCAAGAGAAGAGGTAAATGGCAAGAAGATGTTTGGGGT |
|  |  |
| *Au_674_CPR* | ATGGCTCAATTGGATACTTTGGATGTTGTTGTGTTGGCAGTTCTATTGGCAGGTTCTATTGCATACTTCACCAAAGGTACTTTGTGGGCTGTTGCTAAAGACCCTTATGCTTCCAGTGGTACTGCTATGAATGGTGCTGCTAAAGCTGGAAAGTCAAGAAATATTATCGAGAAGATGGATGAAACCGGAAAGAACTGTGTCATCTTCTACGGTAGTCAAACTGGTACGGCTGAAGATTACGCTTCCAGATTAGCTAAAGAAGGTTCACAAAGATTCGGTTTGAAAACTATGGTCGCTGATTTGGAAGAATACGACTTTGAGAACTTGGATAAGTTTCCAGAGGACAAAGTTGCTTTCTTCGTCTTAGCAACATACGGTGAAGGTGAACCAACTGATAATGCTGTTGAGTTCTACCAGTTCTTCACTGGTGAAGATGTTTCATTTGAAGATGGTGCATCTGCTGAAGATAAGCCATTGTCTAGTCTGAAATACGTCACATTCGGCTTGGGTAATAACACTTATGAACACTACAATGCTATGGTTAGACAAGTTGATGCAGCTTTGACCAAATTGGGTGCTCAAAGAATCGGTTCTGCTGGTGAGGGTGATGATGGTGCTGGTACTATGGAAGAGGACTTTCTAGCTTGGAAAGAACCAATGTGGGCTGCTTTGTCTGAAGCTATGGGTTTGCAAGAAAGAGAATCTAGCTATGAACCAGTGTTCTCAGTTGTCGAAGATGAATCTCTTTCTCCAGAAGATGATTCTGTTTACTTAGGTGAACCTACTAAAGGTCATTTAGAAGGTCAAGCTAAAGGTCCATATTCTGCTCATAATCCATTCATCGCTCCAATCGTTGAGTCCAGAGAATTGTTTACTGTCAAAGATAGAAACTGTCTACACATGGAGATTAGCATTGCTGGTTCTAATTTGACTTACCAAACTGGTGATCATATCGCTGTTTGGCCAACTAATGCTGGTGCTGAAGTTGATAGATTCTTGCAAGTGTTTGGTTTAGAAGAGAAACGTCATTCTGTGATTAACATCAAGGGTATTGATGTTACCGCAAAGGTTCCAATTCCAACTCCAACTACTTATGATGCAGCAGTTAGATACTATATGGAAGTCTGTGCACCTGTTTCCAGACAATTCGTTTCCACATTAGCTGCATTTGCTCCAGATGAAGAAAGTAAAGCTGAAATCGTCAGATTGGGTTCTGACAAAGATTATTTCCATGAGAAGATCACTAACCAATGCTTCAACATTGCTCAAGCTCTTCAATCTATCACCTCTAAGCAATTCTCTAATGTTCCATTTAGCTTGTTGATCGAAGGTTTGAATAAGATCCAACCAAGATACTACTCCATTTCCAGTTCATCTTTGGTTCAGAAAGACAAAATCTCCATTACTGCTGTTGTCGAATCTGTCAGATTGCCAGGTGCTACTCATGTTGTTAAAGGCGTTACTACTAATTACTTGCTGGCATTGAAGCAGAAACAGAATGGTGATCCATCTCCAGATCCACATGGTCAAACATACGCTATCACTGGTCCAAGAAATAAGTACGATGGTATCCATGTACCTGTTCATGTCAGACATTCCAACTTTAAGTTGCCATCTGATCCATCAAGACCAATCATCATGGTAGGTCCAGGTACAGGCGTTGCTCCATTTAGAGGCTTTATCCAAGAAAGAGCTGCATTAGCAGCTAGAGGTGAGAATGTTGGTCCAACTGTTCTATTCTTCGGTTGTAGAAATAGAGAGGAGGACTTTCTGTACAAAGACGAATGGAAAGTATTCCAAGATCAATTAGGTGATAACCTGAAGTTAATTACCGCCTTTAGTAGAGAAGGTTCTCAGAAAGTCTACGTTCAACATAGATTGAGAGAACATGCTGAACTAGTCTCTGATTTGCTAAAGCAGAAAGCTACTTTCTATGTTTGTGGTGATGCTGCTAACATGGCTAGAGAGGTTAACTTGGTCTTAGGTCAAATTATTGCAGCTCAAAGAGGTTTGCCAGCTGAGAAAGGTGAAGAAATGGTGAAACACATGAGATCCAGTGGTTCTTATCAAGAAGATGTCTGGTCT |
|  |  |
| *AuCYB5* | ATGTCTGCTGGCAAAGAGTTCACCTTCAAAGAAGTTGCTGAACACAATACAAAGAAAGACCTTTACTTGGTTGTTCATGATAAGGTGTATGATTGTTCTTCATTCGTTGATGAACATCCAGGTGGTGAAGAAGTTCTTCTTGATGTCGGTGGTCAAGATTCCACTGAGGCTTTCGAAGATGTTGGTCATTCCGATGAAGCTAGAGAGATATTGGAAGGTTTGTTGGTTGGTTCTCTGAAGAGATTACCAGGTGATCCAGCTCCAAAAGTCCATGCTCAAACTAGTTCTGACTCTTCATCTTCAAGCTCTTCAAGTGGTTTCGGTATTGGTTTGTACGCTATTCTATTGATTGGTGGTGCTGCTGCTTATGGTGCATTTCAGTACTTGCAAGCATCAGCAGCAGCTGAACAAGCA |

**Table S4.** ^1^H (600 MHz) and ^13^C NMR (150 MHz) data of 5-OH-21-CHO-OphF and OphU in CDC_l3_

|  | **5-OH-21-CHO-OphF** | |  | **OphU** | |
| --- | --- | --- | --- | --- | --- |
|  | *δ*_C_ | *δ*_H_, *J* (Hz) |  | *δ*_C_ | *δ*_H_, *J* (Hz) |
| 1 | 36.3 | 1.08 dd (14,5, 12.6), 1.64 s |  | 35.3 | 1.04 dd (14.7, 12.6), 1.59 d (3.5) |
| 2 | 51.6 | 2.31 d (3.3) |  | 51.0 | 2.31 d (3.3) |
| 3 | 81.4 |  |  | 81.5 |  |
| 4 | 54.0 | 1.87 dd (15.2, 3.8), 2.68 dd (15.2, 7.6) |  | 53.6 | 1.86 dd (15.1, 4.1), 2.66 dd (15.1,7.6) |
| 5 | 73.3 | 4.87 ddd (7.6, 3.8, 1.2) |  | 73.1 | 4.91 dd (7.8, 4.0) |
| 6 | 50.8 | 3.02 d (9.5) |  | 50.6 | 3.01 d (9.6) |
| 7 | 142.6 |  |  | 142.2 |  |
| 8 | 163.9 | 7.01 m |  | 163.9 | 6.93 t (8.6) |
| 9 | 25.0 | 2.42 m |  | 25.6 | 2.24 td (12.0, 9.0), 2.88 dd (12.7, 8.3) |
| 10 | 53.6 | 1.62 m |  | 54.0 | 1.55 m |
| 11 | 44.2 |  |  | 44.1 |  |
| 12 | 42.9 | 1.38 m, 1.44 m |  | 42.9 | 1.38 m |
| 13 | 23.2 | 1.42 m, 1.54 m |  | 26.7 | 1.58 d (7.1), 1.78 m |
| 14 | 45.6 | 2.34 m |  | 47.4 | 2.09 m |
| 15 | 33.1 | 1.65 m |  | 35.9 | 2.72 td (9.5, 6.4) |
| 16 | 37.2 | 1.15 m, 1.23 m |  | 137.7 | 5.21 t (9.8) |
| 17 | 26.6 | 1.66 m, 1.97 m |  | 122.3 | 6.03 m |
| 18 | 124.8 | 5.10 dddd (9.9, 7.1, 2.7, 1.3) |  | 120.3 | 6.00 m |
| 19 | 131.5 |  |  | 135.9 |  |
| 20 | 26.3 | 1.28 s |  | 26.5 | 1.25 s |
| 21 | 198.3 CHO | 9.26 d (1.0) |  | 198.3 | 9.26 s |
| 22 | 19.0 | 0.94 s |  | 18.6 | 0.99 s |
| 23 | 16.8 | 0.79 d (6.8) |  | 20.6 | 0.91 d (6.6) |
| 24 | 25.9 | 1.71 d (1.4) |  | 18.3 | 1.74 s |
| 25 | 17.8 | 1.60 d (1.3) |  | 26.7 | 1.82 s |
